# Targeting the epigenome to reinvigorate T cells for cancer immunotherapy

**DOI:** 10.1186/s40779-023-00496-2

**Published:** 2023-12-04

**Authors:** Dian Xiong, Lu Zhang, Zhi-Jun Sun

**Affiliations:** 1 State Key Laboratory of Oral and Maxillofacial Reconstruction and Regeneration, Key Laboratory of Oral Biomedicine Ministry of Education, Hubei Key Laboratory of Stomatology, School & Hospital of Stomatology, Frontier Science Center for Immunology and Metabolism, Wuhan University, Wuhan, 430079 China; 2 Department of Oral Maxillofacial-Head Neck Oncology, School and and Hospital of Stomatology, Wuhan University, Wuhan, 430079 China

**Keywords:** Epigenetic therapy, Immune checkpoint blockade, Combination therapy, T cell exhaustion, Immune macroenvironment, Spatial immune contexture, Immunometabolism, Cancer microbiome

## Abstract

Cancer immunotherapy using immune-checkpoint inhibitors (ICIs) has revolutionized the field of cancer treatment; however, ICI efficacy is constrained by progressive dysfunction of CD8^+^ tumor-infiltrating lymphocytes (TILs), which is termed T cell exhaustion. This process is driven by diverse extrinsic factors across heterogeneous tumor immune microenvironment (TIME). Simultaneously, tumorigenesis entails robust reshaping of the epigenetic landscape, potentially instigating T cell exhaustion. In this review, we summarize the epigenetic mechanisms governing tumor microenvironmental cues leading to T cell exhaustion, and discuss therapeutic potential of targeting epigenetic regulators for immunotherapies. Finally, we outline conceptual and technical advances in developing potential treatment paradigms involving immunostimulatory agents and epigenetic therapies.

## Background

The success of immune checkpoint blockade (ICB), now established as the fourth pillar of anticancer therapy, hinges on the concept that immune tolerance of cancer is orchestrated through immune checkpoint-mediated T cell exhaustion. Thus, the removal of such restraint promises to activate cytolytic tumor-specific T cells (TSTs) and bolster anticancer immunity. T cell exhaustion is characterized by transcriptional [1, 2], metabolic [3] and epigenetic programs [4], along with thymocyte selection-associated HMG box (TOX)-dependency [5, 6] and sustained expression of inhibitory receptors [e.g., programmed death-1 (PD-1) [7] and T cell immunoglobulin domain and mucin domain-3 (TIM-3) [8, 9]]. This state is also marked by a hierarchical loss of effector function and proliferative potential, compromising immune surveillance efficacy. Therefore, T cell exhaustion may be exploited by cancer cells for immune evasion, with PD-1 expression on tumor-infiltrating lymphocytes (TILs) indicating poor patient survival in diverse tumor types [10–15]. While T cell-based immunotherapies such as ICB, show clinical successes, disparities in patient outcomes underscore the imperative for investigating ICB resistance mechanisms [16].

**Fig. 1:**
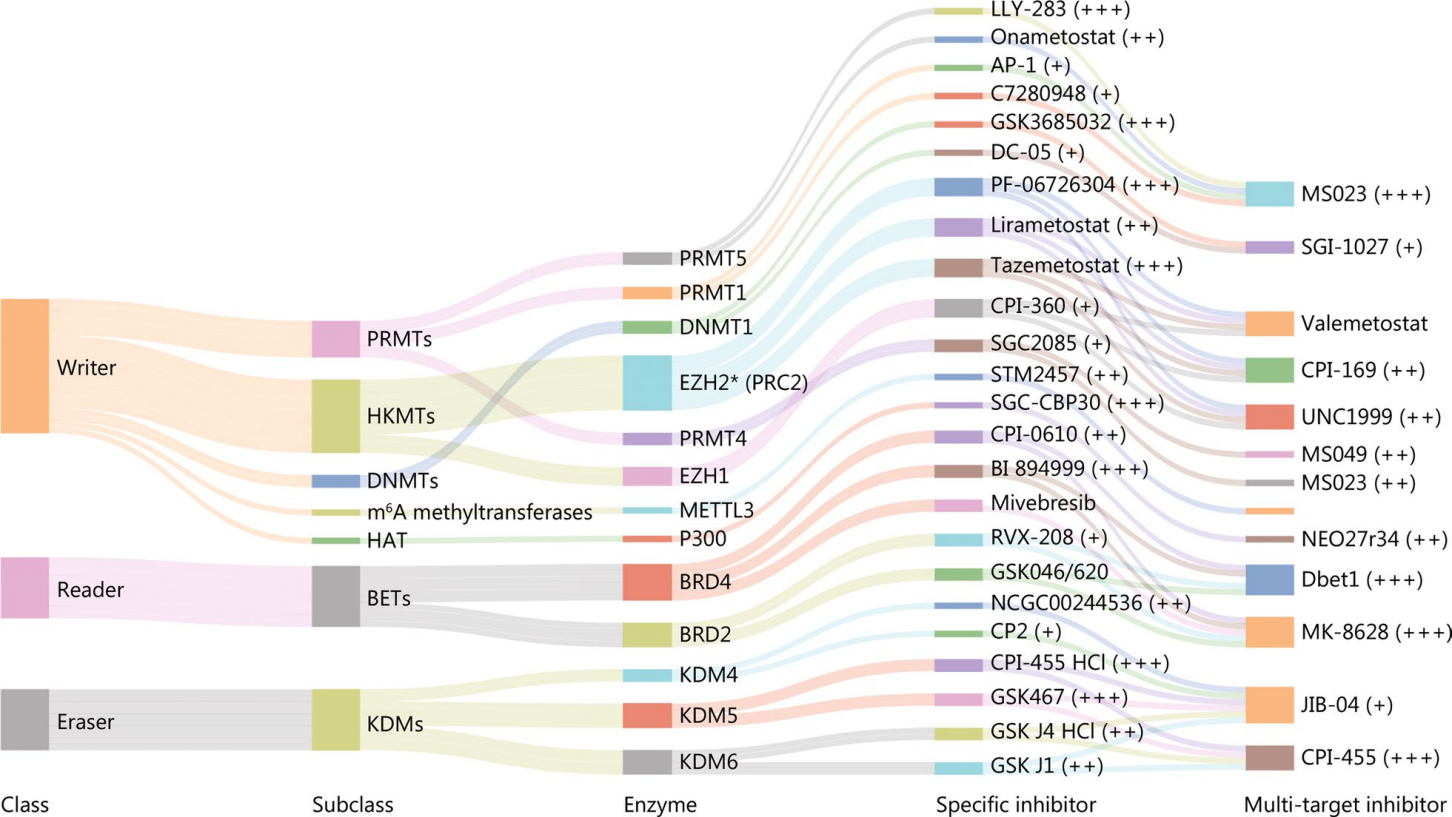
Therapeutic targeting of the epigenetic regulators with specific and multi-target small molecule inhibitors. *as part of the polycomb repressive complex 2 (PRC2) epigenetic complex; ‘^+^’ indicates inhibitor-enzyme selectivity, and for EMCs with multiple substrates their highest selectivity is listed. PRMTs protein arginine methyltransferases, HKMTs histone lysine methyltransferases, HAT histone acetyltransferase, BETs bromodomain and extra-terminal domains, EZH1 enhancer of zeste homolog 1, EZH2 enhancer of zeste homolog 2, METTL3 methyltransferase like-3, BRD bromodomain-containing protein, KDMs lysine demethylases

The intricate mechanisms of T cell exhaustion in cancer immunotherapy, including emerging roles of epigenetic reprogramming, have been discussed in recent seminal reviews [17–19]. Epigenetic therapy, also known as epitherapy, utilizes epigenetic-modifying compounds (EMCs) (Fig. 1) or CRISPR-based epigenome editing [20–22] to modulate epigenetic machineries, including DNA methylation, histone methylation, acetylation and acylation [collectively known as post-translational modifications (PTMs)], as well as chromatin-remodelers and non-coding RNAs (ncRNAs). This regulates the activity of epigenetic enzymes termed “writers”, “readers”, and “erasers” that deposit, remove, or bind to epigenetic marks, respectively. Thus far, certain inhibitors of DNA methyltransferases (DNMTs) and histone deacetylases (HDACs), either alone or in combination, have been approved by the Food and Drug Adminstration (FDA) for the treatment of various malignancies [23], while selective inhibitors of the acetyl-lysine reader–bromodomain and extra-terminal domain (BET) proteins are being tested in clinical trials [24]. Fully understanding the epigenetic regulatory mechanisms underlying T cell exhaustion is critical to harness the potential of epitherapies.

Recent studies on ICB mechanisms identify tumor-draining lymph node (TdLN) as the primary site of action for immune-checkpoint inhibitors (ICIs) [25–27], which refocuses attention on the role of systemic T cells and the immune macroenvironment [28, 29]. Meanwhile, epitherapy could reshape both the tumor immune microenvironment (TIME) and the immune macroenvironment through modulating spatial immune organization, tumor immunogenicity, immunosuppressive cell populations, cancer microbiome, and immunometabolism. This approach offers potential in reinstating endogenous antitumor immunity as validated by pre-clinical and clinical studies [26–33]. Therefore, elucidating how epigenetic mechanisms modulate the TIME and immune macroenvironment is pivotal for advancing ICI-based combinational therapeutic paradigms.

In this review, we summarize the epigenetic regulation of environmental cues driving T cell exhaustion and their therapeutic targeting through ICI-epigenetic therapies. We integrate findings from the latest studies on the spatiotemporal dynamics of T cell exhaustion during ICI treatment, demonstrating that the main site of action for ICIs extends beyond the TIME. Consequently, targeting potent immunosuppressive factors in both the immune macroenvironment and the TIME is critical to surmounting ICI resistance. Building on this, we detail the epigenetic machineries involved in resistance mechanisms. Finally, we discuss recent clinical trial advancements and offer perspectives for guiding the development of combinatorial strategies that include epigenetic modulators, ICIs, and other immunostimulatory agents.

## Exhausted T cells in cancer immunity

Subsets of TILs with an “exhaustion profile” have been recognized, akin to those in chronic lymphocytic choriomeningitis virus (LCMV) infection models [34–38], positioning T cell exhaustion as a significant concern in the field of onco-immunology. Immune checkpoint proteins programmed death-ligand 1 (PD-L1), cytotoxic T lymphocyte-associated antigen-4 (CTLA-4), alongside arginase 1 (ARG1), TIM-3, and estrogen receptor-binding fragment-associated antigen 9 (EBAG9), potentially encapsulated in exosomes, mediate systemic immune suppression and local tumor progression [39]. The lineage relationship between T cell populations induced by viral infection or cancer is being revealed by single-cell technologies pairing transcriptomics and epigenomics with T cell receptor (TCR) sequencing and clonotyping analysis [40, 41]. A plethora of studies have characterized several TST subtypes, each with a varying degree of resemblance to memory T (T_MEM_) cells, effector T (T_EFF_) cells, and terminally exhausted T (T_EX_) cells [42–44], present both inside and outside the TIME.

Adding to the complexity, migration of fresh TSTs external to the TIME in response to ICB could replace exhausted TILs. This process, termed "clonal replacement" [40], is supported by the clinical observation that only a minimal overlap of TCR clonotypes exists between TILs derived from pre- and post-ICI treatment tumor samples: 84% TCRs in the post-treatment group are clonotypes highlighting the expansion of systemic TSTs responding to ICB [40]. This replenishment of ICB-responsive TIME CD8^+^ T-cell pool from the periphery (i.e., normal adjacent tissue, peripheral blood [45], and TdLN [26]) has been observed in multiple tumors [46–48] and credited with the successful prediction of ICI clinical response for PD-L1 inhibitors [31]. A subset of T cell factor 1 (TCF1)^+^TOX^−^ TSTs in TdLNs (accounting for around 40% of total TSTs in TdLNs at 8 weeks post-tumor induction) displays an epigenome distinct from their TCF1^+^TOX^+^ precursor exhausted T (T_PEX_) cells [27]. These TCF1^+^TOX^−^ TSTs are featured with accessible chromatin regions (ACRs) in binding sites of several members of the E26 transformation-specific (ETS) and runt-related (Runx) transcription factors (TFs) families, mirroring canonical T_MEM_; hence this subset is termed TdLN-derived tumor-specific memory cells or TdLN-T_TSM_. This TdLN-T_TSM_ cell population exhibits a more memory-like phenotype with about 150-fold expansion vs. 40-fold for T_PEX_ cells upon antigen re-encounter and heightened responsiveness to ICB, hence being regarded as bona fide responders to ICB.

Therefore, within the immune macroenvironment, TdLN-T_TSM_ cells undergo ICB-induced differentiation into TdLN-T_PEX_ cells, which subsequently clonally supplant the terminally exhausted CD69^+^Ly108^−^ T_EX_ cells in the TIME [27, 43]. This model highlights the role of both the systemic and local immune environment in fostering and deploying ICB-induced cytolytic TSTs.

## Epigenetic machineries and T cell exhaustion

**Fig. 2:**
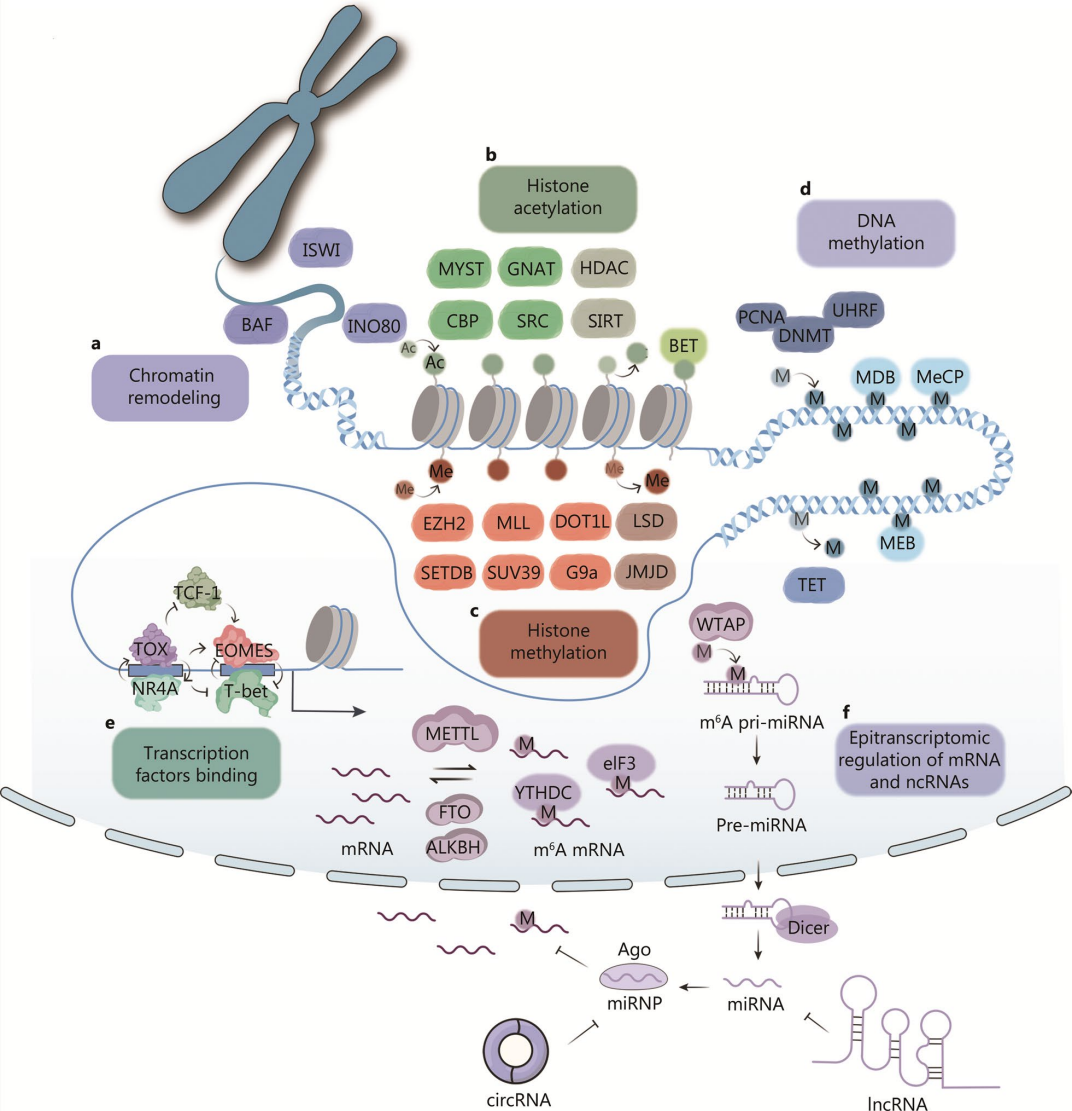
Epigenetic modifications can occur at different levels, including chromatin remodeling, histone modification, DNA methylation, transcription factor binding, and epitranscriptomic regulation of mRNAs and ncRNAs. **a** Chromatin remodeling is mediated by ATP-dependent complexes, such as brahma-associated factor (BAF), inositol requiring 80 (INO80), and imitation switch (ISWI), which could alter DNA accessibility. **b** Histone acetylation is balanced between "writer" enzymes, such as MYST (MOZ, Ybf2/Sas3, Sas2, and TIP60), cAMP-response element binding protein-binding proteins (CBP), general control non-repressed 5 protein-related N-acetyltransferases (GNAT), and proto-oncogene tyrosine-protein kinase Src (SRC) protein families, and "erasers", such as HDAC and silent mating type information regulation 2 homolog 1 (SIRT) family proteins. Histone acetylation can be recognized by "readers", such as the BET family, which are associated with transcriptional activation. **c** Histone methylation is maintained by writers, such as SET, disruptor of telomeric silencing 1-like (DOT1L), and myeloid/lymphoid leukemia (MLL) protein families, and erasers such as lysine-specific demethylase (LSD) and Jumonji C domain-containing (JMJD) families. Histone methylation could be recognized by readers, such as plant homeodomain (PHD) finger proteins. **d** DNA methylation is regulated by DNMT and ten-eleven translocation (TET) proteins and can be recognized by readers such as methyl-CpG-binding domain protein 2 (MDB) and methyl CpG binding protein (MeCP). **e** Transcription factors (TFs) compete with nucleosomes for DNA access and could be recruited by various epigenetic modifiers. The interplay between transcription factors and epigenetic regulators is highly dynamic and complex, with TFs often working in concert with epigenetic modifiers to regulate gene expression. **f** Epitranscriptomic regulation of mRNAs and non-coding RNAs (ncRNAs), such as N^6^-methyladenosine (m^6^A) and 5-methylcytosine (m^5^C) modification, play crucial roles in gene expression and cellular differentiation. RNA modifications can be recognized by reader proteins, such as YTH domain-containing proteins, which regulate mRNA stability, translation, and splicing. miRNP miRNA ribonucleoprotein complex, pre-miRNA precursor miRNA, pri-miRNA primary miRNA, HDAC histone deacetylase, BET bromodomain and extra-terminal motif protein, PCNA proliferating cell nuclear antigen, UHRF ubiquitin-like with PHD and ring finger domains, DNMT DNA methyltransferase, EZH2 enhancer of zeste homolog 2, SETDB SET domain bifurcated histone lysine methyltransferase, TCF-1 transcription factor 1, TOX thymocyte selection-associated high mobility group box, EOMES eomesodermin, NR4A nuclear receptor subfamily 4 group A, M nucleotide methylation, Me histone methylation, METLL methyltransferase-like family proteins, eIF3 eukaryotic translation initiation factor 3, YTHDC YTH domain containing, FTO fat mass and obesity-associated protein, ALKBH AlkB homolog, MEB methyl-CpG binding protein, Ago argonaute proteins

We have outlined the cellular and molecular progression of T cell exhaustion programs in the context of cancer immunotherapy. T cell exhaustion is both established and maintained by finely tuned transcriptional programs that are governed by the “epigenetic landscape” (Fig. 2).

To establish a cell-state specific epigenetic landscape, a DNA sequence-specific deployment of TFs is regulated by their physical access to chromatinized DNA**—**the chromatin accessibility [49]. The accessibility to chromatin (the core structural component of which is the nucleosome) is further modulated by epigenetic modifications such as DNA methylation, histones PTMs, the composition (e.g., the acidic patch [50]) of nucleosomes, and the competition between TFs and nucleosomes for open DNA sequences (i.e., nucleosome occupancy [51]). Moreover, higher-order chromatin conformations, molded by chromatin remodelers [52], provide an extra level of epigenetic control beyond chromatin accessibility, as certain DNA regulatory elements are positioned hundreds of kilobases away from their target genes and require three-dimensional (3D) chromatin folding to be brought together [53]. Additional epigenetic control can be introduced by the presence of multiple PTMs within one nucleosome, and when both activating (e.g., H3K27ac and H3K4me3) and repressive PTMs (e.g., H3K27me3 and H3K9me3) are present, such an epigenetic mark is called "bivalent". Bivalent gene loci signify a transcriptionally poised state [54], accounting for the rapid initiation of gene expression in naïve T cell activation and T_MEM_ cells upon antigen re-encounter. Multivalent histone marks also exist, and together with all forms of PTMs constitute the "histone code" [50], as part of the larger "epigenetic code" [55, 56]. This genetic model reflects how epigenetic information can be encoded in the form of epigenetic modifications that can be maintained and recognized by reader enzymes.

### DNA methylation

DNA methylation at the promoter region represents one of the best-understood epigenetic silencing pathways, involving the transfer of a methyl group from S-adenosyl methionine to the cytosine residue of DNA, particularly at CpG dinucleotides. Its maintenance is dependent upon "writer" DNMTs, "eraser" TETs, and "reader" methyl-CpG binding domain proteins (MBDs).

Small molecule inhibitors targeting enzymes regulating DNA methylation are widely available, with azacitidine (AZA) and decitabine (DAC) being FDA-approved DNMT inhibitors (DNMTi) for treating myelodysplastic syndromes (MDS) [57]. Other DNMTis include RG108 and SGI-1027 which block DNMT’s binding to DNA. As for TET inhibitors, 5-carboxy-8-hydroxyquinoline (5-CHQ), corticosterone methyl oxidase-like protein 1 (CMO1) inhibitors, and pyridine-2,4-dicarboxylic acid derivatives are in development, targeting the TET enzymatic site or inhibiting the conversion of 5-methylcytosine to 5-hydroxymethylcytosine (5hmC). Optimizing TET inhibitors for clinical efficacy and safety remains a research focus. Notably, isocitrate dehydrogenase 1 (IDH1) and IDH2 mutations could lead to α-ketoglutarate (α-KG)-derived oncometabolite two-hydroxyglutarate (2HG) which antagonizes the TET and the Jumanji family of histone demethylases. Therefore, IDH1 and IDH2 inhibitors (ivosidenib and enasidenib) would regulate both DNA and histone demethylation, and have been approved by the FDA for the treatment of relapsed or refractory acute myeloid leukemia (AML) [58].

### Histone acetylation

Histone acetylation, entails adding an acetyl group to lysine residues on histone tails, a key mechanism for regulating gene expression in eukaryotic cells. The balance between histone acetyltransferases (HATs) "writers" and HDACs "erasers" plays a critical role in maintaining proper gene expression levels and cellular function, modulating chromatin structure by neutralizing histone charges and opening the chromatin structure, making the DNA more accessible for TFs as the *cis*-acting effects of histone PTMs. On the other hand, HDACs extract acetyl groups from lysine residues on histone tails, leading to compact chromatin structure and decreased gene expression. There are several HAT families, including general control non-repressed 5 protein-related N-acetyltransferases (GNAT), MYST (MOZ, Ybf2/Sas3, Sas2, and TIP60) and p300/cAMP-response element binding protein-binding proteins (CBP). For HDACs, they are classified into four classes based on phylogenetic relationship, protein structure, and subcellular localization: Class I (HDAC1, 2, 3, and 8), Class II [Class IIa (HDAC4, 5, 7, and 9) and Class IIb (HDAC6 and 10)], Class III (also known as sirtuins), and Class IV (HDAC11) [59].

Several small molecule inhibitors of histone deacetylases (HDACi) have been approved by the FDA for the treatment of various blood malignancies, such as cutaneous and peripheral T-cell lymphoma and multiple myeloma. These HDACis are vorinostat, romidepsin, belinostat, and panobinostat [60]. Other potent HDACis, including entinostat (MS-275), mocetinostat (MGCD0103), abexinostat (PCI-24781), and resminostat (4SC-201), are currently undergoing clinical trials for the treatment of breast cancer, Hodgkin's lymphoma, lymphoma, hepatocellular carcinoma (HCC), and colorectal cancer [60]. Furthermore, trichostatin A and valproic acid (VPA) are also utilized for treating other diseases. CUDCs are a class of bifunctional inhibitors that target HDAC and other proteins, and they have shown promising antitumor effects in preclinical models and clinical trials for various solid tumors and hematologic malignancies. Examples include CUDC-101, which inhibits HDAC1, epidermal growth factor receptor (EGFR), and human epidermal growth factor receptor 2 (HER2) and has been effective in preclinical models of lymphoma and solid tumors, and CUDC-907, which has demonstrated a significant response rate in relapsed/refractory diffuse large B-cell lymphoma patients [61, 62].

The use of proteolysis-targeting chimeras (PROTACs) represents promising avenue for achieving sustained inhibition and degradation of HDACs. PROTACs comprise a ligand that binds to the target protein, an E3 ligase recognition moiety, and a connecting linker. Once the ligand binds to the target protein, the E3 ligase mediates ubiquitination and subsequent degradation of the target protein. Several HDAC-targeted PROTACs have been developed, including those targeting HDAC6, which utilizes a selective HDAC6 inhibitor, nexturastat A, conjugated to pomalidomide as cereblon (CRBN)128 [63]. Notably, the PROTACs NP8 and NH2 degraded HDAC6 without impacting other HDAC types. The reversible degradation of HDAC6 by PROTACs was demonstrated by the recovery of HDAC6 three hours after removal of the PROTACs [64].

### Histone methylation

Histone methylation on lysine and arginine residues in the N-terminal tails is regulated by histone methylation transferases (HMTs) and "eraser" lysine demethylases (KDMs), similar to histone acetylation. HMTs, which acquire methyl groups from S-adenosylmethionine (SAM) like DNMTs, could be classified into several families including the SET domain-containing proteins [e.g., enhancer of zeste homolog 2 (EZH2), SET domain bifurcated histone lysine methyltransferase 1 (SETDB1), and Su(Var)3–9 homolog 1 (SUV39H1)], DOT1L, and PR domain-containing proteins (e.g., PRMT1-8 and G9a [65]). These enzymes may target different sites and catalyze distinct methylation modification patterns, either activating [e.g., H3K4me1/2/3 by SET1A/B, H3K36me2/3 by SET domain containing 2 (SETD2), and H3K79me1/2/3 by DOT1L] or repressive (e.g., H3K9me1/2/3 by SUV39H1/2, G9a and SETDB1, and H3K27me1/2/3 by EZH1/2 and MLL2/3). On the other hand, lysine-specific KDMs are epigenetic enzymes that catalyze the removal of methylation marks from histone lysine and arginine residue respectively. Different subfamilies of KDMs (KDM1-7) can be categorized into flavin adenine dinucleotide (FAD)-dependent lysine-specific demethylases (LSDs) and α-KG-dependent Jumonji C domain-containing (JMJD) families [66]. Selective KDM small molecule inhibitors including KDM1A (LSD1) inhibitor ORY-1001 for acute lymphoblastic leukemia (ALL) and small-cell lung cancer, KDM4 inhibitor IOX1 for glioblastoma, KDM5 inhibitor CPI-455 for ALL, and KDM6 inhibitor GSK-J4 for various malignancies, are under clinical evaluation.

### Chromatin binding factors

The differentiation of cell types (e.g., T_EFF_ and T_MEM_) and cell states (e.g., exhaustion) hinges on the complex interactions between chromatin-binding factors and nucleosomes that cooperatively regulate chromatin accessibility and 3D conformation. An in vivo Perturb-seq study revealed that chromatin remodeling complexes INO80 and brahma-associated factor (BAF) are crucial to an early commitment to exhaustion in T cells [67]. In exhausted chimeric antigen receptor (CAR)-T cells, chromatin conformation capture coupled with Hi-C chromatin immunoprecipitation (HiChIP) revealed that chromatin loopings are anchored at the promoter region of key exhaustion genes (i.e., *PDCD1*, *HAVCR2*, *LAG3*, *TIGIT*, and *TOX*), suggesting that extensive chromatin remodeling may occur in the development of CAR T_EX_ cells [68].

Targeting strategies against chromatin remodeling complexes include PROTACs developed as degraders of the BAF adenosine triphosphatase (ATPase) subunits SMARCA2 (SWI/SNF-related matrix-associated actin-dependent regulator of chromatin subfamily A, member 2) and SMARCA4. Detailed biophysical investigation and high-resolution ternary complex crystal structures enabled rational and optimized design of ACBI1, a potent and cooperative degrader of SMARCA2, SMARCA4 and PBRM1 [69]. ACBI1 demonstrated anti-proliferative effects and induced cell death caused by SMARCA2 depletion in SMARCA4 mutant cancer cells, along with impacts in AML cells reliant on SMARCA4 ATPase activity.

Additional approaches include macrolactams like BD-98 that dissociate AT-rich interaction domain 1A (ARID1A)-containing BAF complexes from chromatin and inhibitors like BI-9564 that disrupt the bromodomain-containing protein 9 (BRD9) subunit [70, 71]. Targeting strategies have also been identified against other chromatin remodeling complexes. For example, the imitation switch (ISWI) ATPase can be inhibited by flavonol ignorin to disrupt nucleosome spacing [72]. Clinically available inhibitors exist against chromatin reader proteins like BET bromodomains (JQ1, iBET, CPI-0610) and plant homeodomain (PHD) fingers (I-CBP112). Overall, major advances have been made in targeting chromatin remodeling complexes to subtly yet precisely manipulate the regulatory chromatin landscape.

### ncRNAs

Small ncRNAs and long ncRNAs (lncRNAs) have emerged as key contributors to genome stability and epigenetic memory [73]. RNA-mediated gene silencing involves an RNA scaffold, serving as platform for recruiting histone lysine methyltransferase (HKMT) and DNMT complexes [i.e., polycomb and ubiquitin-like with PHD and RING finger domains 1 (UHRF1)-DNMT1] to mediate heterochromatin formation. In particular, small ncRNAs such as the endogenous miRNA or exogenous small interfering RNA (siRNA) assemble with the Argonaute (Ago) protein to form an RNA–protein complex (RISC) central to RNA interference (RNAi) mechanisms [74]. RNAi has been shown to perform gene silencing both on the translational level (TGS) targeting histones or on the post-translational level (PTGS), targeting mRNAs [75]. Specific miRNAs facilitate PTGS in T cell exhaustion: miR-31 enforces a sustained expression of type I interferon by antagonizing mRNA *Ppp6c* which is a negative regulator of interferon signaling, thereby increasing PD-1 surface expression on T cells [76]; miR-155 promotes the expansion of T_EX_ subset through inhibiting AP-1 family TF FOS-like antigen 2 (FOSL2) [77]; further, miR-29a ameliorates T cell exhaustion by inhibiting translational circuits including inflammatory and TCR signaling, as well as ribosomal biogenesis [78]. Apart from small ncRNAs and lncRNAs, circular RNAs (circRNAs) represent another class of ncRNAs capable of post-translational gene regulation [79, 80], through modulating the alternative splicing of pre-mRNAs or "sponging" certain families of miRNAs. circRNAs including circHMGB2, circ_0020710, and circTRPS1 have been identified as mediators of T cell exhaustion via respective sponging of miR-181a-5p, miR-370-3p, and miR-141-3p, leading to T_EX_ biology [81–88] (Table 1).

**Table 1:** circRNAs associated with CD8^+^ T cell exhaustion and immunosuppression

Cancer type	circRNA	Mechanism	References
NSCLC	circIGF2BP3^a^, circHMGB2	Increases PKP3 expression via miR-328-3p and miR-3173-5p sponging, upregulating PD-L1 in tumor cells and inactivating the type I interferon response by inhibition of PRMT4 via miR-181a-5p sponging	[81]
	circRNA-002178^a^	Upregulates PD-L1 expression in tumor cells and PD-1 expression in T cells respectively via miR-34a and miR-28-5p sponging	[84]
	circIGF2BP3^b^	Increases PKP3 expression via miR-328-3p and miR-3173-5p sponging, upregulating PD-L1 in tumor cells	[85]
NPC	circBART2.2	Promotes transcription of PD-L1 through binding to the helicase domain of RIG-I, thereby activating transcription factors IRF3 and NF-κB	[86]
PC	circ_0046523	Upregulates PD-L1 expression in PC cells via miR-148a-3p sponging	[87]
HCC	circMET	Upregulates CXCL10 level through miR-30-5p sponging to abrogate Snail/DPP4-mediated immunosuppression	[88]
Melanoma	circ_0020710	Upregulates CXCL12 level via miR-370-3p sponging	[82]
BCa	circTRPS1^b^	Upregulates PD-1 expression through enhancing GLS1-mediated glutamine metabolism via miR-141-3p sponging	[83]

^a^Available as exosome-derived circRNAs; ^b^Further regulated by m^6^A modification; *NSCLC* non-small-cell lung cancer, *NPC* nasopharyngeal carcinoma, *PC* pancreatic cancer, *BCa* bladder cancer, *PKP3* plakophilin 3, *RIG* iretinoic acid-inducible gene I, *IRF3* interferon regulatory factor 3, *NF-κB* nuclear factor kappa-B, *CXCL10* C-X-C motif chemokine ligand 10, *GLS1* glutaminase 1, *HCC* hepatocellular carcinoma, *PD-L1* programmed death-ligand 1, *PRMT4* protein arginine methyltransferases 4

Similar to the covalent modifications of histones and DNA, RNA modifications such as N^6^-methyladenosine (m^6^A), N^1^-methyladenosine (m^1^A), pseudouridine (Ψ) and adenosine-to-inosine (A-to-I) editing have regulatory roles in immune and cancer cell biology, as excellently reviewed in these articles [89, 90]. T cell activation is accompanied by the upregulation of tRNA methyltransferase 6 (TRMT6), which modifies a specific subset of early-expressed transfer RNAs (tRNAs) to enhance translation efficiency and synthesis of MYC and other functional proteins that promote T cell activation and proliferation [91].

Strategies for targeting ncRNAs include direct oligonucleotide-based inhibitors and miRNA mimics [92]. Antisense oligonucleotides (ASOs) can redirect splicing by binding intronic regulatory sequences, while also targeting expanded repetitive sequences in diseases like myotonic dystrophy [93]. lncRNAs present challenges for small molecule targeting due to poor conservation and unknown mechanisms, however they can be targeted by ASOs and RNAi [94]. Recent advances demonstrate targeting miRNA precursors, including a neomycin-nucleobase-amino acid conjugate inhibiting pre-miR-21 processing [95]. Likewise, a ribonuclease targeting chimera (RIBOTAC) recruiting RNase L achieved selective degradation of pre-miR-21, inhibiting breast cancer metastasis in mice [96]. Conjugating the RNA-binding drug dovitinib to this RIBOTAC scaffold enhanced inherent RNA-targeting activity while decreasing protein target effects [97]. Overall, these oligonucleotide-centered techniques and conjugates demonstrate potential for modulating regulatory ncRNAs.

Taken together, the establishment and maintenance of an epigenetic landscape that define the cellular state and identity of exhausted CD8^+^ T cells are enforced by specialized epigenetic regulators such as writers, erasers and readers of PTMs, DNMTs, chromatin remodelers, heterochromatin-associated complexes as well as ncRNAs. Fueled by technological advancements in single-cell transcriptomics, clonotyping, and epigenomics, further discoveries regarding the role of epigenetics in the diverse array of T cell states and subtypes hold great potential for the development of epitherapies aimed at preventing or reversing T cell exhaustion.

## Epigenetic regulation of T cell exhaustion drivers

Cancer has been increasingly recognized as a systemic disease with global immune ramifications [28, 98]. In tumor-burdened host, extensive reorganization of the "immune macroenvironment" is observed, with a peripheral expansion of immunosuppressive cell types such as myeloid-derived suppressor cells (MDSCs), tolerogenic dendritic cells (DCs) and Treg cells [29, 99, 100]. This further leads to intratumoral accumulation of immunosuppressive cells in the TIME, exacerbating the TIL exhaustion and conferring resistance to T cell-based immunotherapies [101].

**Fig. 3:**
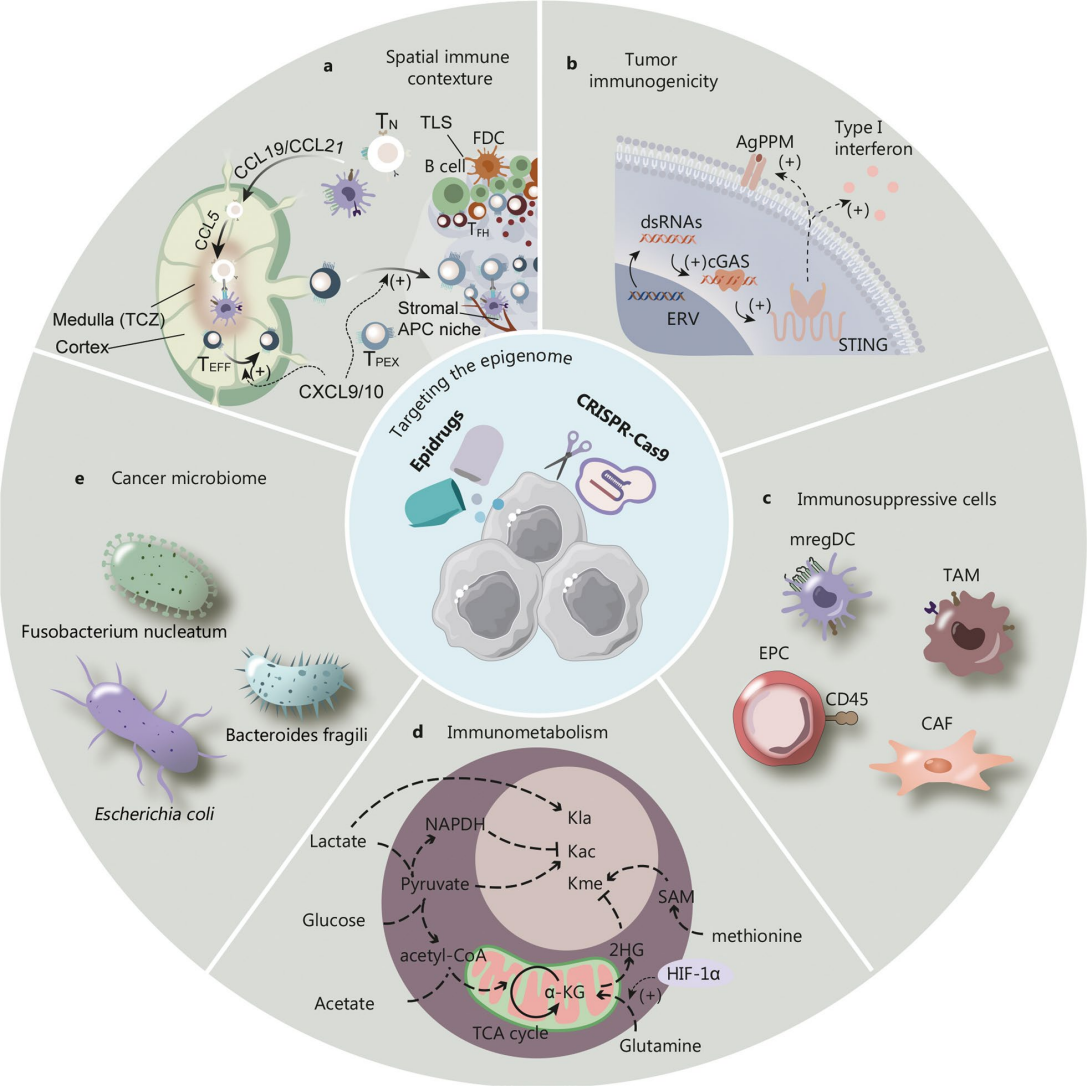
Targeting the epigenetic regulation of extrinsic drivers of T cell exhaustion via epidrugs and CRISPR-based epigenome editing. **a** Epigenetic modulation of chemokines could direct the formation and coordination of spatial immune contexture. T_N_ and DCs migrate into the TdLN via CCR7-CCL19/21 chemokine axis as part of the cancer-immunity cycle, together with CXCL9/10-dependent trafficking of T_EFF_ and T_PEX_ cells from the TdLN to the TIME. Within TdLN CCL5 gradient mediates homing of TN cells to the T cell zone (TCZ) in the medulla for priming by antigen presenting cells (APCs), while similar APC niches exist in the stromal compartments of the TIME, crucial to ICI-elicited antitumor immunity. Within the TIME tertiary lymphoid structures (TLSs) consist of follicular dendritic cells (FDCs), B cells, follicular T helper (T_FH_) cells and CXCL13^+^ dysfunctional T cells. This subset is speculated to mediate the formation of TLSs via releasing CXCL13, which could be enhanced by epigenetic therapies. **b** Epigenetic upregulation of tumor immunogenicity could be achieved via increased expression of endogenous retroviruses (ERVs) and their transcription to produce double-strand RNAs (dsRNAs), which are sensed by pattern recognition receptors such as the cGAS (cyclic guanosine monophosphate-adenosine monophosphate synthase)-STING (stimulator of interferon genes) pathways, eventually resulting in upregulation of antigen processing and presentation machineries (AgPPM) and release of type I interferon. **c** Epigenetic reprogramming of immunosuppressive cells in the TIME and the immune macroenvironment could revitalize antitumor immunity. **d** Level of extracellular metabolites in the TIME could impact histone modifications (Kla, lysine lactylation; Kme, lysine methylation; Kac, lysine acetylation) through providing cofactors and donor groups to epigenetic enzymes. **e** Epigenetic reprogramming mediates the microbiomic modulation of anticancer immunity. CAF cancer-associated fibroblast, CCL19 C–C motif chemokine ligand 19, CCR7 C–C motif chemokine receptor 7, EPC erythroid progenitor cells, mregDC mature DC enriched in immunoregulatory molecules, TAM tumor-associated macrophage, acetyl-CoA acetyl coenzyme A, T_N_ naive T cells, T_PEX_ precursor exhausted T cells, TCZ T cell zone, SAM S-adenosylmethionine, HIF-1α hypoxia-inducible factor 1-alpha, CXCL9/10 C-X-C motif chemokine ligand 9/10

Moreover, tumor-induced perturbations in TdLN-DCs could hamper cross-presentation of tumor antigen and CD28 co-stimulation of T cells [102]. This has profound implications for ICB therapies, as the systemic nature of ICI-elicited anticancer response has been unveiled by mass spectrometry-based profiling of global immune dynamics [103, 104]. The critical interaction of PD-1^+^ TSTs and PD-L1^+^CD103^+^cDC1 in the TdLNs is both a mediator and indicator of ICB efficacy [17], and the enhancement of TdLN-T cell priming with CD40 agonist also enhances ICB-driven de novo TSTs response in pre-clinical studies and clinical trials [105]. Therefore, the simultaneous reversal of tumor-driven systemic immune perturbations is critical to unleashing ICI-induced anticancer immunity. This section will delve into how epitherapies might address this need, with a summary on the epigenetic regulation of systemic immune macroenvironment and the spatial coordination of antitumor immunity between TIME and TdLNs. Further, we summarize other factors contributing to TIL exhaustion, from dampened tumor immunogenicity to immunosuppressive cells in the systemic immune environment and the TIME, and consider the role of epigenetic reprogramming in conferring the effects of immunometabolism and microbiome on anticancer immunity (Fig. 3).

### Epigenetic modulation of the immune macroenvironment

#### Chemokines

Stimulator of interferon genes (STING) upregulation in tumor APCs triggers chemokine expression, notably C-X-C motif chemokine ligand 9 (CXCL9) and CXCL10 by DCs, and CXCL10 and CXCL11 by tumor-associated macrophages (TAMs), folstering intratumoral T cell trafficking [106, 107]. Chemokines orchestrates immune cell migration and localization in tissues. In the cancer-immunity cycle, the C–C motif chemokine receptor 7 (CCR7)-CCL19/CCL21 axis governs the entry of cDC1 and T_N_ into the TdLN, while the C-X-C motif chemokine receptor 3 (CXCR3)-CXCL9/CXCL10 axis regulates the egress of T helper 1 (Th1) cells and CD8^+^ TSTs from the TdLN into the TIME. The Th1-type chemokines CXCL9/CXCL10 can be silenced by DNMT and EZH2 via increased methylation at the promoter region [32]. This silencing leads to T cell exclusion and immune evasion, echoing similar findings [108, 109]. Moreover, other subunits of the polycomb complex apart from EZH2 [i.e., suz12 polycomb repressive complex 2 subunit (SUZ12), and embryonic ectoderm development (EED)] are also inversely associated with Th1-type chemokines and patient survival in colon cancer [110], and similarly HDAC in lung adenocarcinoma [111]. The recruitment of CD8^+^ TSTs can be further mediated by the CCR5-CCL5 axis, which can be increased by sequential use of DNMTi and HDACi which result in MYC depletion [112]. As MYC is a key TF regulating early T cell exhaustion, its inhibition improves T cell trafficking into the TIME and augment tumor immunogenicity by promoting endogenous retroviruse (ERV) transcription [113]. In parallel, circMET and circ_0020710 contribute to T cell exhaustion by upregulating CXCL10 and CXCL12 respectively via sponging tumor suppressive miRNAs [82, 97]. Therefore, targeting regulatory circRNAs along with modulation of MYC hold promise for relieving T cell exhaustion by tuning chemokine-mediated cell trafficking and immunosuppression.

#### Immune contexture

The "immune contexture" within tumors is characterized by the chemokine milieu which regulates the phenotype and function of immune cells by dictating their localization and cellular communications in the TIME [114]. Advances in spatial transcriptomics and imaging technologies have highlighted the dynamic interplay of immune cells in the tumor setting [115, 116] Chemokines have emerged as important cues for the establishment of intratumoral immune niches and TLSs, which contribute to the housing of stem-like TCF1^+^ TSTs and the recruitment of functional TSTs. In particular, a group of CXCL13^+^CD8^+^ T cell subset could initiate TLS formation through CXCL13-dependent recruitment of B cells and follicular helper T cells (T_FH_). This subset is further correlated with effective responses to PD-1/PD-L1 ICB therapies [117]. The expression of CXCL13 can also be epigenetically tuned by the combined application of the HDACi entinostat with tumor-targeted delivery of interleukin-12 (IL-12), which achieved an elevated level of CXCL13 and CXCL9. This strategy succeeded in overcoming resistance to PD-1/PD-L1 ICB in tumors harboring MHC-I and interferon γ (IFN-γ) deficiencies [118]. Nonetheless, the role of epigenetic mechanism in the formation of TLS is less well understood, arguing for the design of precise single-cell transcriptomic and epigenomic studies to elucidate TLS biology [119].

In addition to TLS, other immune niches are observed in both tumors and TdLNs. In tumor tissue, perivascular niches [120] and intra-epithelial niches [121] have been identified, sharing the common characteristic of a clustering of stem-like or effector TSTs with APCs. Such localization of TSTs in close proximity with APCs benefits T cell response to ICB and is regulated by the CCR5-CCL5 axis, which recruits naïve CD8^+^ TSTs to tumor antigen-loading cDC1s. Epigenetic regulation of the CCR5-CCL5 axis is mediated by ATP-dependent chromatin remodeling complexes, including SWI/SNF and HATs such as p300 and CBP [101]. Furthermore, the CXCR3-CXCL9/CXCL10 axis regulates the spatial partitioning of CCR7^+^ stem-like T cells vs. CXCR3^+^ T_EFF_ cells. In head and neck tumor tissues, CCR7^+^TCF1^+^ stem-like TSTs preferentially occupy stomal areas, while the CXCR3^+^PD1^+^ intermediate TST subsets with effector function and dysfunctional TSTs infiltrate the tumor parenchyma [122]. Conversely, in draining lymph nodes (dLNs) CXCR3^+^ T_EFF_ cells chemotaxis to the peripheral cortex while CCR7^+^ stem-like TSTs home to the T cell zone (TCZ) in dLN medulla [123]. Therefore, the interactions between chemokines and their cognate receptors coordinate effective systemic antitumor immunity at the spatial and cellular level, presenting therapeutic targets for epigenetic interventions.

### Epigenetic modulation of tumor immunogenicity

While PD-1/PD-L1 inhibitors do not exclusively target the TIME, their reactivation effects on resident TSTs occur alongside the emergence of TST clones. Clinically, the dual contribution of TSTs is unique per individual [43], explaining the variability in ICI treatment responses. For patients with severe intratumoral T cell exhaustion, ICI therapy likely depends on systemic immune responses for tumor elimination [27]. To initiate a systemic antitumor response, a cancer-immunity cycle is essential: tumor APCs, such as cDC1 cells, must present tumor neoantigens in the dLNs, where TST priming and activation occur [28]. Activated TSTs then infiltrate the TIME, leading to tumor destruction by effective TILs. Epigenetic modulation dynamically regulates this cycle, offering chances for EMCs to enhance immune response and tumor targeting in combination with various therapies. Mechanistic studies suggest that DNMTi, EZH2i, and HDACi upregulate tumor antigens and antigen presentation pathways [124, 125], enhancing the synergy between ICIs, cancer vaccines [126], and CAR T/NK cells [127, 128]. Furthermore, tumor cell surface receptor expression, like elevated PD-L1 and reduced Fas—which modulate immune suppression and resistance to T cell cytotoxicity—is epigenetically alterable [129]. Specific circular RNAs in various cancers are known to upregulate PD-L1, thereby reducing tumor immunogenicity [93–96]. Intracellularly, the cyclic guanosine monophosphate-adenosine monophosphate (cGAS)-STING signaling pathway is repressed via promoter hypermethylation of the *cGAS* and *STING* genes, and can be rescued by KDM5i and AZA, leading to pro-inflammatory cytokine production, enhanced antigen presentation and tumor immunogenicity [130–132].

### Epigenetic modulation of immunosuppressive cells

In addition to chronic stimulation of TCR signaling, stems from the immunosuppression within the TIME, enforced by cell populations such as TAMs, MDSCs, regulatory DCs, Treg cells, as well as cancer-associated fibroblasts (CAFs) and erythroid progenitor cells (EPCs). These cells diminish T cell function and confer resistance to ICIs, characterized by high surface expression of inhibitory molecules, including PD-L1 for myeloid cells (i.e., TAMs, MDSCs) and CTLA-4 for Treg cells, along with suppressive cytokines including IL-10 and transforming growth factor-beta (TGF-β) [133, 134]. Epigenetic mechanisms are key in determining the differentiation and activity of these immunosuppressive groups.

#### Myeloid suppressive cells: MDSCs, TAMs, and mature DC enriched in immunoregulatory molecules (mregDCs)

The cyclooxygenase-2 (COX2)/microsomal prostaglandin E synthase-1 (mPGES1)/prostaglandin E2 (PGE2) signaling pathway influences PD-L1 expression via DNMT3A [135]. Moreover, signal transducer and activator of transcription (STAT)-dependent expression of the enzymes arginase 1 and inducible nitric oxide synthase (iNOS) as MDSC hallmarks can be attenuated by inhibition of BET-H3K27 acetylation [136]. Transcriptomic profiling of MDSCs indicates DNMT and polycomb gene silencing machineries contribute to immunosuppression [137]. However, the EZH2i GSK126 drives MDSC differentiation in the TIME and resistance to EZH2i treatment in lymphoma, highlighting the need for developing targeted EZH2i, potentially conjugated with cell-specific antibodies [138]. Obesity-related cancer study showed that adipose tissue macrophages, central to obesity-linked inflammation and tumor progression, are reprogrammed in an obesity-associated milieu through altered metabolite exchange, cytokine production, extracellular vesicle content, and gut microbiota metabolites [139]. In TAMs, M1/M2 polarization is regulated by a lineage-determining TF PU.1 which displaces nucleosomes to establish the activating H3K4me1 marks on DNA sequences that guide macrophage polarization [140].

Another recently characterized group of mregDCs, along with immature DCs in the TIME, contribute to immune tolerance and TIL exhaustion [141, 142]. This group of CCR7^+^PD-L1^+^ DCs exhibit dual functionality: they are immunosuppressive through PD-L1 expression and pro-inflammatory via IL-12 secretion, although IL-4 signaling can inhibit the latter mechanism. Notably, blocking IL-4 augments IL-12 production by mregDCs and enlarges the TIL repertoire [141]. The impact of IL-4 on DCs is mediated by TET-dependent DNA demethylation. Consequently, TET enzymes, together with SETD1A—an H3K4 methylation "writer"—are regulators of DC differentiation. The TF early growth response 2 (EGR2) could recruit and interact with TET, triggering the differentiation of monocyte-derived DCs [143]. Interestingly, time-course data on DNA methylation and gene expression patterns in DCs in response to infection suggested that gene expression level changes prior to the demethylation programs [144]. This suggests that DNA demethylation may not be imperative to establishing DC-specific transcriptional programs; instead, the role of TF binding to *cis*-acting elements might be more predominant.

#### Treg cells

The ratio between PD-1^+^ T_EFF_ and CD4^+^CD25^+^ Treg cells could predict clinical efficacy of ICB therapy [145], with Treg cells expanded upon PD-1 inhibitor treatment [146]. Similar to T_EX_ subsets, the development and function of Treg cells are programmed by key TF forkhead box P3 (FOXP3) with sustained expression through the demethylation of *Foxp3* locus [147]. Accessibility of the Treg cell-specific demethylation region (TSDR) can be abolished during CRISPR-based *Foxp3* silencing, indicating the essential role of *Foxp3* in regulating TSDR genes [148, 149]. In an in vitro setup, 5-azacytidine mediates the demethylation of TSDR and upregulates FOXP3 expression, abolishing the immunosuppressive function of Treg cells, even resulting in an increase of IL-17^+^FOXP3^+^ “effector" Treg cells [150]. However, contrasting results are reported elsewhere [151] and the net effect of 5-azacytidine on Treg requires more research, considering the important role of 5-azacytidine in the treatment of hematological malignancies and autoimmune disorders [152, 153]. Likewise, the overall activating effect of several HDACis (e.g., inhibitors for HDAC6/9/11 and SIRT1) on Treg is documented [154, 155], while HDAC5 alone may inhibit the suppressive functions of Treg cells [156]. These studies demonstrate the need to develop isoform-specific HDACis for clinical application. CBP/p300, another set of transcriptional co-activators, are known to drive the differentiation of regulatory T cells through both transcriptional and non-transcriptional mechanisms, underscoring the complexity of T_REG_ regulation [157]. The BET-H3K27 acetylation transcriptional activating pathway also contributes to stable expression of FOXP3, and its abrogation suppresses T_REG_ functions [158]. In contrast, the chromatin-modifying enzyme EZH2, critical for the maintenance of T_REG_ identity post-activation, presents a different mechanism of action, with its inhibition leading to a reprogramming of intratumoral T_REG_ cells and enhanced cancer immunity [159, 160]. Notably, unlike its pro-tumor effects on MDSCs, EZH2i disrupt T_REG_ biology and synergizes with anti-CTLA4 ICB in murine models [161].

#### CAFs

In the TIME, apart from immune constituents, CAFs constitute another class of immunosuppressive agents. Originating from normal fibroblasts and mesenchymal precursors such as pericytes and adipocytes, CAFs emerge in response to oncogenic signals within TIME. Similar to T_EX_ subsets, the heterogeneity and plasticity in CAF cell types have been revealed by single-cell RNA-sequencing (scRNA-seq), under the transcriptional regulation by CAF-associated TFs such as STAT3. A combined array analysis of DNA methylation and gene expression in human mesenchymal stem cells (MSCs) pre- and post-tumor co-culture reveals that tumor-induced methylation of STAT3 are pivotal for CAF activation and tumor growth, an effect reversible by 5-azacytidine [162]. Moreover, a STAT3-dependent reprogramming of normal fibroblast into invasive CAFs is driven by leukemia inducible factor (LIF)-induced DNA methylation. In parallel, LIF-induced histone acetylation upregulates DNMT3b-dependent DNA methylation, reinforcing STAT3 signaling [163]. HDAC6-mediated deacetylation also upregulates STAT3 and PGE2/COX2, which worsens the immunosuppressive effects of CAFs [164]. Furthermore, overexpressing high mobility group at-hook 2 (Hmga2) in prostate stromal cells induces CAF formation within the TIME [165]. Taken together, these findings underscore the intricate network of epigenetic modifications, encompassing DNA methylation, PTMs, and chromatin remodeling, that govern CAF development and modulate the TIME. Intriguingly, it has been shown that increased lactate production within the TIME has been linked to elevated α-KG levels in MSCs, initiating their transformation into CAFs [166], highlighting an epigenetic link between metabolic shifts and anticancer immunity.

#### EPCs

EPCs, as immature erythroid progenitors and precursors of red blood cells, proliferate within the immune macroenvironment including dLNs and the spleen, and in the TIME [167]. By generating suppressive cytokines like TGF-β and IL-10, alongside reactive oxygen species (ROS) and PD-L1 expression, EPCs attenuate T cell activity [168–170]. These suppressive pathways parallel those utilized by MDSCs, suggesting that they may be similarly amenable to epigenetic interventions. EPCs are further segregated into CD45^+^ and CD45^−^ subsets; the CD45^+^ EPCs, marking an earlier erythroid differentiation stage, comprise over 40% of the EPC population in tumor-bearing mice and are chiefly accountable for immunosuppression [169, 171]. Thus, enhancing erythropoiesis could potentially alleviate T cell suppression in both the macroenvironment and the TIME.

Erythroid differentiation arrest and the resultant EPC accumulation might stem from epigenetic misregulation. Erythroblasts exhibit stage-specific phenotypes, transcriptomes, and epigenetic profiles [172, 173]. EPC maturation is marked by TET-mediated demethylation and chromatin restructuring, which facilitate enhancer-promoter interactions [174, 175]. In one study, VPA was found to drive the differentiation of stem-like CD34^+^ cord blood cells by increasing H3 acetylation of promoters for erythroid-specific genes [176]. Additionally, *Dnmt1* and *Ezh2* are identified among genes associated with erythropoiesis [176], and it’s been shown that EZH2-mediated epigenetic silencing of the pro-apoptotic *Bim* contributes to erythropoiesis [177]. However, in the terminal stage of erythropoiesis, the accumulation of repressive histone marks (e.g., H3K9me3, H3K27me3, and H4K20me1) mediated by LSD1 [178] and Setd8 [179] as well as the decrease of activating histone mark (e.g., H3K27ac) mediated by HDAC2 [180] and HDAC5 [181] are involved in the terminal maturation of human erythroblasts. These insights underline the potential of epigenetic modulation in erythropoiesis. Approaches to epigenetic therapy should be tailored, with treatment regimens timed to target epigenetic dynamics active at specific erythropoiesis stages, employing agents like selective HDACi, EZH2i, and DNMTi for early-stage intervention.

### Epigenetic reprogramming and immunometabolism

Epigenetic reprogramming significantly influences T cell immunometabolism, a key determinant of their functionality within the TIME. Growing evidence has shown that exhausted T cells exhibit metabolic insufficiency with suppressed mitochondrial respiration and glycolysis [182, 183]. Both inefficient nutrients (e.g., glucose and methionine) and detrimental metabolites (e.g., lactate and glutamine) may contribute to the commitment to the exhaustion phenotype by altering the epigenetic mechanisms regulating T cell development [184]. Specifically, glucose scarcity impedes glycolytic flux, leading to a shortfall in acetyl coenzyme A (acetyl-CoA), which is the acetyl source for histone acetylation. This deficiency is evidenced by reduced H3K9ac at the *Ifng* locus in T cells, correlating with diminished IFN-γ production, whereas acetate supplementation restores IFN-γ production even in glucose-restricted conditions [185]. Similarly, elevated extracellular lactate level can disrupt cellular redox balance [186], as indicated by an increased NADH/NAD^+^ ratio, which in turn can stifle T cell activity by inhibiting aerobic glycolysis and the function of the NAD^+^-dependent HDAC, SIRT1—a key enzyme supporting the development of T cells capable of enhanced tumor control [187]. Nonetheless, the role of SIRT1 within the TIME is complex, as it also influences the function of Treg cells and MDSCs [188].

Methylation also has an essential role in conferring the effects of immunometabolism. The activity of TET and JMJD3 is dependent on α-KG, a product of glutamine catabolism, and α-KG-dependent H3K27 demethylation is involved in the dysfunction of TSTs [189]. In addition, the hypoxia-inducible factor 1α (HIF-1α)-dependent accumulation of the α-KG-derived oncometabolite 2HG antagonizes the α-KG-dependent TET functions and leads to global histone and DNA methylation in adoptively transferred CD8^+^ T cells, promoting their in vivo persistence [190]. Conversely, increased methionine uptake leads to the accumulation of SAM as the methyl donor for all methylation programs, including histones and nucleic acids. The downregulation of SAM is associated with loss of the H3K79me2 mark at STAT5 promoter and impaired T cell immunity [191]. Likewise, methionine restriction resulted in reduced SAM level and demethylation of H3K4me3, thereby disrupting the differentiation of inflammatory Th17 cells [192]. Furthermore, increased methionine uptake permits histone and nucleic methylation programs, including the methylation of the m^6^A nucleotide in RNA mediated by the methyltransferase like-3 (METTL3) methyltransferase. Such methylation schemes participate in the regulation of T-cell differentiation programs and the TIME [193]. Interestingly, high level of extracellular potassium, despite suppressing T_EFF_ programs by restricting nutrient uptake, induces histone deacetylation at the exhaustion loci and promote T cell stemness in the TIME [194]. Therefore, epigenetic mechanisms including PTMs and nucleic acid methylation underpin the modulatory effects of cell metabolism on the phenotypes and function of TIME-TSTs. Future research is needed to elucidate possible epigenetic regulation on certain exhaustion-related immunometabolic pathways such as mitochondrial stress, endoplasmic reticulum stress, and hypoxia [195, 196].

### Epigenetic reprogramming and cancer microbiome

The human microbiome has been acknowledged as a hallmark of cancer [197], plays a pivotal role in carcinogenesis, therapeutic responses, and antitumor immunity [198]. The gut and intratumoral microbiota specific to tumor types and individuals may tailor individual’s response to ICIs, highlighting the microbiota-mediated modulation of antitumor immunity. Evidence suggests a correlation between microbiota composition and positive responses to ICIs, with unique microbial signatures differentiating responders from non-responders in ICB treatments, as revealed by integrated 16S rRNA and metagenomic shotgun sequencing [199]. Mechanistically, the effector function of CD8^+^ TSTs and DC activation is stimulated by oral administration of *Bifidobacterium* alone or in combination with anti-PD-L1 [200]. Similarly, a consortium of 11 strains of bacteria from healthy human gut was capable of inducing IFN-γ production in CD8^+^ T cells and MHC-I expression in CD103^+^ DCs [201]. A relative abundance of *Akkermansia muciniphila* (*A. muciniphila*) was associated with improved ICI efficacy in the patients of non-small-cell lung cancer (NSCLC), renal cell carcinoma, and urothelial carcinoma [202]. Correspondingly germ-free (GF) mice receiving fecal microbiota transplant (FMT) from non-responder FMT displayed resistance to ICB and was reversed by supplementation of *A. muciniphila* which increased CXCR3^+^CD4^+^ T cells and decreased Treg cells in the TIME [202]. Additional immune-activating mechanisms for microbiota include activation of pro-inflammatory bioactive molecules [e.g., Toll-like receptors (TLRs) [203] and STING], facilitating the formation of intratumoral TLS [204], and increasing tumor immunogenicity through coating tumor cells with microbial peptides [205].

However, microbial-TLR interactions can also encourage the infiltration of immunosuppressive cells in pancreatic cancer (PC), enhancing tumor immunosuppression. Microbiome dysbiosis could drive cancer progression through epigenetic modulation [206]. Genome-wide bisulfite sequencing of GF and conventionally raised mice revealed that exposure to microbiota-induced TET2/3-dependent aberrant methylation programs in response to acute inflammation, underlying the carcinogenesis of colon tumor in colitis. Consequently, genetic depletion of TET2/3 restores intestinal homeostasis [207]. In addition, oncometabolites produced by microbes are critical to conferring the epigenetic-modifying effects of microbiota. For instance, colonization of butyrate-producing bacteria strains leads to elevated histone acetylation levels in colorectal adenocarcinomas. In immune cells, microbe-derived short-chain fatty acids (SCFAs) butyrate and propionate could potentiate the generation of Treg cells by inhibiting HDAC activity [208]. Nonetheless, the direct effects of microbiota on modifying the epigenetic landscape of T cell exhaustion are less well documented. This gap in understanding may explain the challenges in using microbiota composition as reliable ICI response biomarkers, further complicated by the variability of the human microbiome [209].

## Insights from clinical trials combining epitherapies and immunotherapies

As T cell exhaustion is increasingly recognized as both a cause and consequence of high tumor burden [210], the concurrent use of epigenetic modulators and tumor burden-reducing immunotherapies may potentiate therapeutic synergy. While DNMTi AZA and DAC upregulate tumor antigens and antigen presentation components [57, 125], HDACi vorinostat and romidepsin promote immunostimulation of TAMs [140]. Additionally, the EZH2i tazemetostat is being evaluated for enhancing T cell responses by curtailing Treg cells [161]. Emerging BET inhibitors may also mitigate exhaustion by downregulating immunosuppressive pathways in MDSCs [136]. Combinational epi-immunotherapy approach could relieve tumor-imposed immunosuppression and reinvigorate endogenous antitumor immunity. Carefully designed clinical trials will be critical to validate and optimize synergistic combinations tailored to specific cancer types and immune contexts.

Since 2002, several small molecule EMCs alone or in combination have been approved by the FDA for the treatment of various hematological malignancies such as AML, MDS, and T-cell lymphomas [23]. However, their combinational schemes with immunotherapies have failed on average to demonstrate improved clinical efficacies in the setting of hematological cancers [211, 212]. Nonetheless, their performances in solid tumors are more promising and have been investigated in numerous phase II and I/II trials [213–215]. This disparity could be due to the following reasons. First, intrinsic and extrinsic mechanisms of resistance in T-cell and non-T-cell compartments contribute to a diminished response to ICIs in patients with leukemia [216]. Second, leukemic blasts could interact with circulating T cells through direct contact and bystander effects [216]. In this way, leukemic blasts rather than localized solid tumor cells are more proximate to circulating TSTs, thereby promoting exhaustion and senescence. Given the importance of circulating TSTs in the replacement of intratumoral TSTs and ICI response, this suppressive effect could particularly undermine the benefits of combination ICI therapies.

The integration of epigenetic therapy with adoptive cell modalities, such as CAR T cells, encountered a setback with the cessation of a phase I trial combining azacytidine and NKR-2 (NCT03612739) at the behest of the sponsor. However, clustered regularly interspaced short palindromic repeats and clustered regularly interspaced short palindromic repeats (CRISPR)-associated protein 9 (CRISPR-Cas9) genome editing has facilitated direct modifications to the T cell epigenome, enabling the deletion of epigenetic regulators like TET2 or the insertion of TFs such as c-Jun and basic leucine zipper ATF-Like transcription factor (BATF) [217–219]. A critical role of exhaustion for CAR T cell persistence has been established in CAR T cell therapy [220–222]. Study suggests that when challenged by the same immunosuppressive TIME, both adoptive CAR and endogenous CD8^+^ T cells display similar patterns of exhaustion with comparable transcriptional and epigenetic rewiring [68]. Notably, CAR T cells display an increased propensity to exhaustion, due to tonic signaling from the synthetic TCR even in the absence of antigen. In line, a transient cessation of such signaling could phenotypically revive exhausted CAR T cells through EZH2-dependent epigenetic remodeling [222]. This reflects the need for applying a molecular "brake" in the design of CARs, mimicking how the low expression of PD-1 helps to maintain TCF-1^+^ T_PEX_ identity [7] and how TOX promotes intratumoral persistence of TSTs [6]. An alternative approach could be to target the specific epigenetic landscape of exhaustion through either direct epigenetic therapies or modifications in CAR T cell engineering to bolster function before reinfusion [223]. Additionally, the application of other immunostimulatory agents, such as oncolytic viruses (OVs) and immunogenic chemotherapy regimens, has been explored alongside epigenetic therapies, as detailed in recent reviews [224, 225].

## Conclusions and perspectives

The exhaustion of tumor-reactive CD8^+^ TILs confers resistance to cancer immunotherapies, underscoring the need to elucidate the developmental cues driving T cell exhaustion for therapeutic targeting. Elements in the immune macroenvironment and the TIME are recognized as mediators of ICB-induced T cell rejuvenation, with the discovery of ICB-responding TST subsets in both peripheral and intratumoral immune niches [27, 28]. Among them, the TdLN-TTSM subset is being recognized as the bona fide responder to ICB [27], aligning with clonal replacement model [40, 48] and dual origins for ICB-induced TST expansion within the TIME [48].

Epigenetic regulators orchestrate programs that modulate chromatin dynamics, acting as intrinsic switches for cellular fate and functional state at the molecular level. DNMTs and TETs control DNA methylation, whereas PTM-associated writers, erasers, and readers alter chromatin structure and accessibility [54]. ncRNAs, modulated by RNA modifications like m^6^A and m^5^C, regulate gene expression and interact with epigenetic modifiers to effect gene silencing [84]. Chromatin remodelers manipulate 3D interactions between promoters and distal regulatory elements [49], and metabolites serve as cofactors or substrates for epigenetic enzymes, conferring immunometabolic effects [187]. The intricate synergy between TFs and epigenetic machinery intrinsically shapes T cell fates and responds to environmental cues sustaining T cell functionality. Thus, a strategy targeting intrinsic and extrinsic factors of T cell exhaustion may enhance ICB-induced T cell revitalization.

**Fig. 4:**
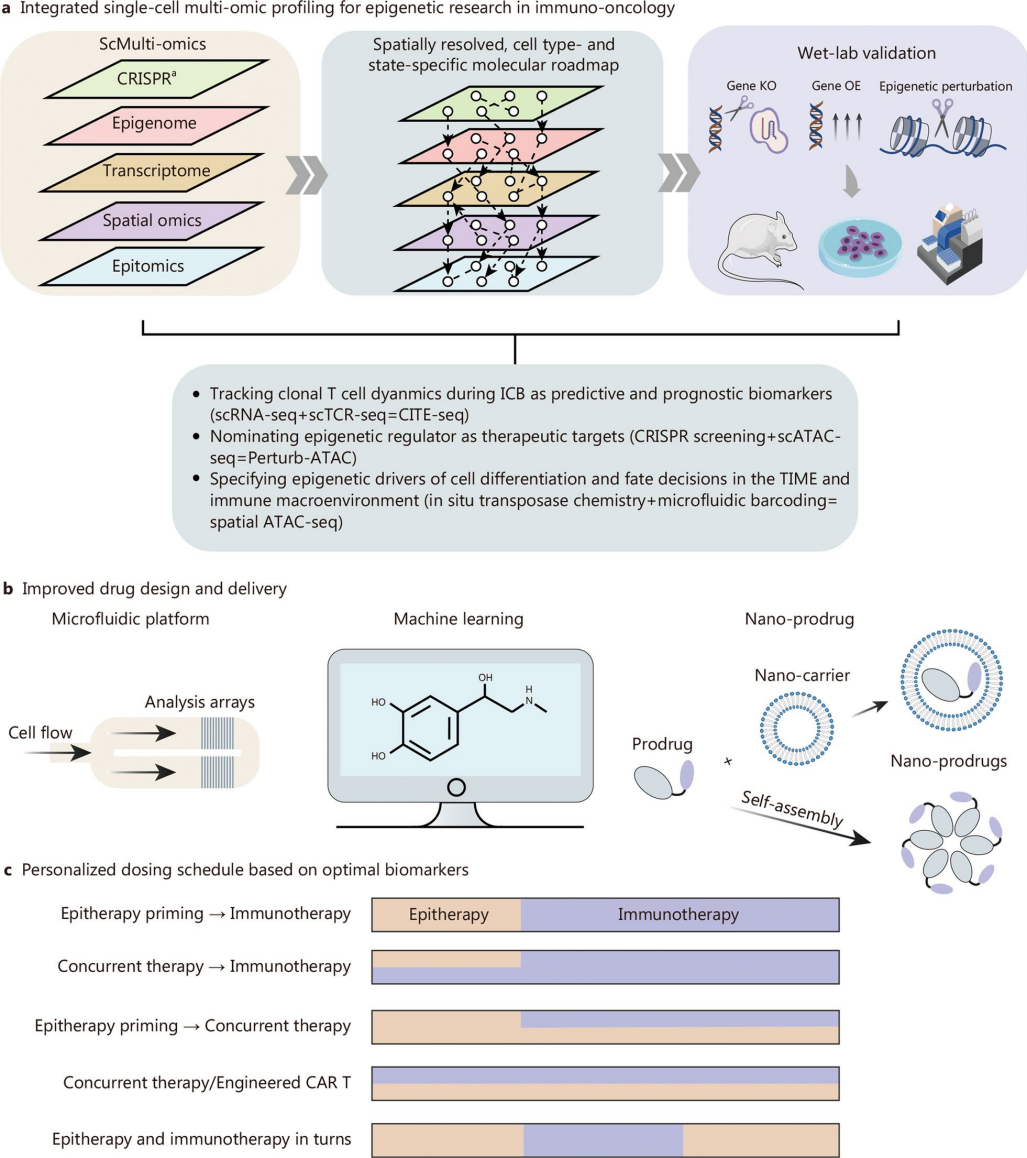
Conceptual and technical advances in the development of immunotherapy-synergized epitherapy. **a** Single-cell multi-omics could be applied to studying the epigenetic regulation of antitumor immunity for designing epigenetic-immunotherapy. Identification of biomarkers on TSTs independent of T cell exhaustion is crucial for assessing the overall ICB efficacy in eliciting antitumor immunity. Such process could be assisted via CITE-seq integrating scRNA-seq and scTCR-seq. Likewise, discovery of epigenetic regulators for therapeutic targeting could be facilitated by Perturb-seq integrating CRISPR screening and scATAC-seq, while spatial information of epigenetic drivers of cell differentiation and fate decisions could be obtained via Spatial ATAC-seq. **b** The extensive use of conventional epidrugs are limited by poor bioavailability, systemic toxicity and lack of selectivity, which could be potentially avoided by advanced drug design and testing platforms and nano-delivery methods. **c** By identifying specific and sensitive biomarkers, individualized epitherapy could be designed with tailored dosing schedules. CAR chimeric antigen receptor, CITE-seq cellular indexing of transcriptomes and epitopes by sequencing, CRISPR clustered regularly interspaced short palindromic repeats, Perturb-seq CRISPR-mediated genetic perturbations with single-cell RNA sequencing, scMulti-omics single-cell multi-omics, scATAC-seq single-cell assay for transposase-accessible chromatin by sequencing, scTCR-seq single-cell T cell receptor sequencing, KO knock out, OE overexpression, ICB immune checkpoint blockade, scRNA-seq single-cell RNA sequencing, CITE-seq cellular indexing of transcriptomes and epitopes by sequencing, scATAC-seq single-cell assay for transposase-accessible chromatin using sequencing, TIME tumor immune microenvironment, ATAC-seq assay for transposase-accessible chromatin using sequencing, CAR chimeric antigen receptor, TSTs tumor-specific T cells

However, the clinical performance of such combinational strategy in hematological malignancies is so far insufficient to warrant further design of phase III trials, while in solid tumors a number of phase II trials are ongoing. Addressing specific challenges is paramount before these combinational treatments can meet the demands posed by current immunotherapies (Fig. 4).

First, limited spatiotemporal resolution in tracking heterogeneous T cell epigenome dynamics during immunotherapy constrains our understanding of their cell fate decisions. However, recent advancements such as spatial ATAC-seq have begun to address these limitations [226–230]. Studies that analyze the spatial organization of diverse CD8^+^ T cell subtypes with unique epigenomic landscapes could significantly enhance our comprehension of the orchestration and establishment of systemic antitumor immunity at both cellular and tissue levels. Single-cell sequencing technologies have provided deep insights into disease pathogenesis, paving the way for accurate diagnostic and therapeutic approaches [226]. Techniques like single-cell cleavage under targets and tagmentation (scCUT&Tag) and the related spatial-CUT&Tag enable high-resolution, genome-wide mapping of chromatin modifications and TFs, unraveling potential epigenetic drivers of immune cell differentiation and tumorigenesis [227–229]. Spatial-CUT&Tag has been successfully benchmarked in the profiling of spatially resolved (i.e., with spatial coordinates) chromatin accessibility for tissue sections including mouse embryos, human central nervous systems, and tonsils [230]. The selection of these tissues highlights the power of spatial epigenomics in deciphering developmental biologies, encompassing organogenesis, immunogenesis and tumorigenesis [230]. Given that epigenetic mechanisms are intrinsic regulators for cell identity, cell state, and fate decisions, insights gained from spatial epigenomic mapping would help us elucidate mechanisms underlying the mobilization of antitumor immunity by immunotherapies at both molecular and cellular level, in both the TIME (harboring TLSs) and TdLNs.

Second, the profound heterogeneity of the TIME across tumor types poses challenges to the identification of overriding regulatory mechanisms for therapeutic targeting. Within each tumor niche, T cells are influenced by a milieu of diverse immunosuppressive factors with overlapping and distinct effects. Though epigenetic modulators may reinvigorate T cell reactivity, their pleiotropic impacts on the TIME remain poorly defined. This is exemplified by the debated net effect of 5-azacytidine on Treg cells [150, 151], where the same epigenetic drug could unpredictably augment certain immunosuppressive mediators while alleviating others. Furthermore, significant knowledge gaps exist regarding T cell extrinsic cues, exhaustion phenotypes, and their interplay. Therefore, deciphering precise immunosuppressive mechanisms in a customized, patient- and tumor-specific manner is integral before rational application of epigenetic or other immunomodulators. The characterization of predictive biomarkers that allows swift identification of TSTs from bystander T cells [231] ex vivo would benefit such studies and their clinical translation. These markers should simultaneously correlate with antitumor immunity and not themselves targeted by ICBs (hence excludes exhaustion-specific markers), so as to reflect the temporal dynamics of TST response throughout ICB treatment. Liu et al. [48] recently identified CXCL13 as biomarker on both treatment-naïve and treatment-induced TSTs, signifying robust T cell response to ICB. These CXCL13^+^ TSTs can be further divided into one functional KI67^high^STMN1^high^ subset, as well as three dysfunctional subsets including two T_PEX_ (IL7R^+^HAVCR2^−^/GZMK^+^HAVCR2^−^) and one T_EX_ (HAVCR2^+^TOX^+^). Comprehensive integration of high-dimensional single-cell profiling with multiplexed spatial imaging and epigenomic assays could illuminate cell–cell interactions driving T cell dysfunction within the TIME topography [232]. Unraveling these interactions promises to reveal prognostic biomarkers and avenues for enhancing immunotherapy on a mechanistic level.

Finally, although FDA-approved small molecule inhibitors of DNMT, HDAC, and EZH2 are available clinically, their extensive application is limited by poor bioavailability, specificity, and systemic toxicity. Promising avenues for addressing these challenges include advanced drug development platforms that integrate microfluidics and machine learning [233, 234]. These platforms could improve drug specificity and reduce toxicity, enhancing the efficacy of epigenetic drugs. Another promising strategy for enhancing the bioavailability of small molecule compounds is the use of prodrug-based nano-delivery systems. These systems can improve drug solubility, stability, and circulation time, resulting in increased drug delivery to the target site and reduced off-target effects [235, 236]. Well-established predictive markers for therapeutic response and commercially available high-throughput epigenome mapping would also assist the sensitivity and toxicity testing of epidrugs and tailoring of dosing schedule on a personalized basis.

## Data Availability

Not applicable.

## Abbreviations

5-CHQ: 5-Carboxy-8-hydroxyquinoline;
5hmC: 5-Hydroxymethylcytosine
ACR: Accessible chromatin region
AgPPM: Antigen processing and presentation
α-KG: α-Ketoglutarate
APC: Antigen presenting cells
ARG1: Arginase 1
ATPase: Adenosine triphosphatase
BAF: Brahma-associated factor
BCa: Bladder cancer
BET: Bromodomain and extra-terminal domain
BRD: Bromodomain-containing protein
CAFs: Cancer-associated fibroblasts
CCL19: C-C motif chemokine ligand 19
CCR7: C-C motif chemokine receptor 7
CITE-Seq: Cellular indexing of transcriptomes and epitopes by sequencing
circRNAs: Circular RNAs
COX2: Cyclooxygenase-2
COMPASS: Complex proteins associated with Set1
cGAS: Cyclic guanosine monophosphate-adenosine monophosphate synthase
CMO1: Corticosterone methyl oxidase-like protein 1
CRBN128: Cereblon
CRISPR: Clustered regularly interspaced short palindromic repeats
CTA: Cancer/testis antigen
CXCL10: C-X-C motif chemokine ligand 10
dLNs: Draining lymph nodes
DOT1L: Disruptor of telomeric silencing 1-like
DNMT: DNA methyltransferases
EED: Embryonic ectoderm development
EGFR: Epidermal growth factor receptor
EPCs: Erythroid progenitor cells
ETS: E26 transformation-specific
EBAG9: Estrogen receptor-binding fragment-associated antigen 9
ERV: Endogenous retrovirus
EZH1: Enhancer of zeste homolog 1
EZH2: Enhancer of zeste homolog 2
FDA: Food and Drug Administration
FDC: Follicular dendritic cell
FOXP3: Forkhead box P3
GC: Gastric cancer
GF: Germ-free
GLS1: Glutaminase 1
GNAT: General control non-repressed 5 protein-related N-acetyltransferases
HKMTs: Histone lysine methyltransferases
HAT: Histone acetyltransferase
HCC: Hepatocellular carcinoma
Hmga2: High mobility group at-hook 2
HDACs: Histone deacetylases
HER2: Human epidermal growth factor receptor 2
HiChIP: Hi-C chromatin immunoprecipitation
HP1α: Heterochromatin protein 1α
ICB: Immune checkpoint blockade
ICIs: Immune checkpoint inhibitors
IDH1: Isocitrate dehydrogenase 1
IFN-γ: Interferon γ
iNOS: Inducible nitric oxide synthase
INO80: Inositol requiring 80
IRF3: Interferon regulatory factor 3
ISWI: Imitation switch
JMJD: Jumonji C domain-containing
KDMs: Lysine demethylases
LCMV: Lymphocytic choriomeningitis virus
LIF: Leukemia inducible factor
lncRNAs: Long ncRNAs
LSD: Lysine-specific demethylase
LSD1: Lysine-specific demethylase 1
MDSC: Myeloid-derived suppressor cell
METTL3: Methyltransferase like-3
MDS: Myelodysplastic syndromes
MeCP: Methyl CpG binding protein
MSC: Mesenchymal stem cell
MS: Mass spectrometry
MDB: Methyl-CpG-binding domain protein
NF-κB: Nuclear factor kappa-B
NPC: Nasopharyngeal carcinoma
ncRNAs: Non-coding RNAs
NSCLC: Non-small-cell lung cancer
OV: Oncolytic virus
PC: Pancreatic cancer
Perturb-seq: CRISPR-mediated genetic perturbations with single-cell RNA sequencing
PHD: Plant homeodomain
PKP3: Plakophilin 3
PRMTs: Protein arginine methyltransferases
PRC2: Polycomb repressive complex 2
PTMs: Post-translational modifications
RIG I: Retinoic acid-inducible gene I
Runx: Runt-related
SCFA: Short-chain fatty acids
scATAC-seq: Single-cell assay for transposase-accessible chromatin by sequencing
scMulti-Omics: Single-cell multi-omics
scRNA-seq: Single cell rna-sequencing
scTCR-seq: Single-cell T cell receptor sequencing
SETD2: SET domain containing 2
SETDB1: SET domain bifurcated histone lysine methyltransferase 1
SGC: Salivary gland carcinoma
SMARCA2: SWI/SNF-related matrix-associated actin-dependent regulator of chromatin subfamily A, member 2
SRC: Proto-oncogene tyrosine-protein kinase Src
STAT: Signal transducer and activator of transcription
STING: Stimulator of interferon genes
SUZ12: Suz12 polycomb repressive complex 2 subunit
SUV39H1: Su(Var)3–9 homolog 1
TAM: Tumour-associated macrophages
TdLN: Tumor-draining lymph node
TFs: Transcription factors
TGF-β: Transforming growth factor-beta
TIL: Tumour-infiltrating lymphocyte
TIME: Tumor immune microenvironment
TLS: Tertiary lymphoid structure
TSTs: Tumor-specific T cells
TNBC: Triple-negative breast cancer
TET: Ten-eleven translocation
UHRF1: Polycomb and ubiquitin-like with PHD and RING finger domains 1
VPA: Valproic acid

## References

[1] Tsui C, Kretschmer L, Rapelius S, Gabriel SS, Chisanga D, Knöpper K (2022). MYB orchestrates T cell exhaustion and response to checkpoint inhibition. Nature.

[2] Zhu L, Zhou X, Gu M, Kim J, Li Y, Ko CJ (2022). Dapl1 controls NFATc2 activation to regulate CD8^+^ T cell exhaustion and responses in chronic infection and cancer. Nat Cell Biol.

[3] Soto-Heredero G, Desdín-Micó G, Mittelbrunn M (2021). Mitochondrial dysfunction defines T cell exhaustion. Cell Metab.

[4] Collier JL, Weiss SA, Pauken KE, Sen DR, Sharpe AH (2021). Not-so-opposite ends of the spectrum: CD8^+^ T cell dysfunction across chronic infection, cancer and autoimmunity. Nat Immunol.

[5] Alfei F, Kanev K, Hofmann M, Wu M, Ghoneim HE, Roelli P (2019). TOX reinforces the phenotype and longevity of exhausted T cells in chronic viral infection. Nature.

[6] Scott AC, Dündar F, Zumbo P, Chandran SS, Klebanoff CA, Shakiba M (2019). TOX is a critical regulator of tumour-specific T cell differentiation. Nature.

[7] Chen Z, Ji Z, Ngiow SF, Manne S, Cai Z, Huang AC (2019). TCF-1-centered transcriptional network drives an effector versus exhausted CD8 T cell-fate decision. Immunity.

[8] Hudson WH, Gensheimer J, Hashimoto M, Wieland A, Valanparambil RM, Li P (2019). Proliferating transitory T cells with an effector-like transcriptional signature emerge from PD-1^+^ Stem-like CD8^+^ T cells during chronic infection. Immunity.

[9] Im SJ, Hashimoto M, Gerner MY, Lee J, Kissick HT, Burger MC (2016). Defining CD8^+^ T cells that provide the proliferative burst after PD-1 therapy. Nature.

[10] Oliva M, Spreafico A, Taberna M, Alemany L, Coburn B, Mesia R (2019). Immune biomarkers of response to immune-checkpoint inhibitors in head and neck squamous cell carcinoma. Ann Oncol.

[11] de Simone M, Arrigoni A, Rossetti G, Gruarin P, Ranzani V, Politano C (2016). Transcriptional landscape of human tissue lymphocytes unveils uniqueness of tumor-infiltrating T regulatory cells. Immunity.

[12] He Y, Yu H, Rozeboom L, Rivard CJ, Ellison K, Dziadziuszko R (2017). LAG-3 protein expression in non-small cell lung cancer and its relationship with PD-1/PD-L1 and tumor-infiltrating lymphocytes. J Thorac Oncol.

[13] McNiel EA, Tsichlis PN (2017). Analyses of publicly available genomics resources define FGF-2-expressing bladder carcinomas as EMT-prone, proliferative tumors with low mutation rates and high expression of CTLA-4, PD-1 and PD-L1. Signal Transduct Target Ther.

[14] Terry S, Dalban C, Rioux-Leclercq N, Adam J, Meylan M, Buart S (2021). Association of AXL and PD-L1 expression with clinical outcomes in patients with advanced renal cell carcinoma treated with PD-1 blockade. Clin Cancer Res.

[15] Mansfield AS, Roden AC, Peikert T, Sheinin YM, Harrington SM, Krco CJ (2014). B7–H1 expression in malignant pleural mesothelioma is associated with sarcomatoid histology and poor prognosis. J Thorac Oncol.

[16] Morad G, Helmink BA, Sharma P, Wargo JA (2022). Hallmarks of response, resistance, and toxicity to immune checkpoint blockade. Cell.

[17] Huang Y, Jia A, Wang Y, Liu G (2023). CD8^+^ T cell exhaustion in anti-tumour immunity: the new insights for cancer immunotherapy. Immunology.

[18] Wang Q, Qin Y, Li B (2023). CD8^+^ T cell exhaustion and cancer immunotherapy. Cancer Lett.

[19] Zhu W, Li Y, Han M, Jiang J (2023). Regulatory mechanisms and reversal of CD8^+^ T cell exhaustion: a literature review. Biology (Basel).

[20] Nuñez JK, Chen J, Pommier GC, Cogan JZ, Replogle JM, Adriaens C (2021). Genome-wide programmable transcriptional memory by CRISPR-based epigenome editing. Cell.

[21] Nakamura M, Gao Y, Dominguez AA, Qi LS (2021). CRISPR technologies for precise epigenome editing. Nat Cell Biol.

[22] Micevic G, Bosenberg MW, Yan Q (2023). The crossroads of cancer epigenetics and immune checkpoint therapy. Clin Cancer Res.

[23] Lu Y, Chan YT, Tan HY, Li S, Wang N, Feng Y (2020). Epigenetic regulation in human cancer: the potential role of epi-drug in cancer therapy. Mol Cancer.

[24] Topper MJ, Vaz M, Marrone KA, Brahmer JR, Baylin SB (2020). The emerging role of epigenetic therapeutics in immuno-oncology. Nat Rev Clin Oncol.

[25] Dammeijer F, van Gulijk M, Mulder EE, Lukkes M, Klaase L, van den Bosch T (2020). The PD-1/PD-L1-checkpoint restrains T cell immunity in tumor-draining lymph nodes. Cancer Cell.

[26] Connolly KA, Kuchroo M, Venkat A, Khatun A, Wang J, William I, et al. A reservoir of stem-like CD8^+^ T cells in the tumor-draining lymph node preserves the ongoing antitumor immune response. Sci Immunol. 2021;6(64):eabg7836. 10.1126/sciimmunol.abg7836 PMC8593910 34597124

[27] Huang Q, Wu X, Wang Z, Chen X, Wang L, Lu Y (2022). The primordial differentiation of tumor-specific memory CD8^+^ T cells as bona fide responders to PD-1/PD-L1 blockade in draining lymph nodes. Cell.

[28] Hiam-Galvez KJ, Allen BM, Spitzer MH (2021). Systemic immunity in cancer. Nat Rev Cancer.

[29] Allen BM, Hiam KJ, Burnett CE, Venida A, DeBarge R, Tenvooren I (2020). Systemic dysfunction and plasticity of the immune macroenvironment in cancer models. Nat Med.

[30] Wang L, Amoozgar Z, Huang J, Saleh MH, Xing D, Orsulic S (2015). Decitabine enhances lymphocyte migration and function and synergizes with CTLA-4 blockade in a murine ovarian cancer model. Cancer Immunol Res.

[31] Yu G, Wu Y, Wang W, Xu J, Lv X, Cao X (2019). Low-dose decitabine enhances the effect of PD-1 blockade in colorectal cancer with microsatellite stability by re-modulating the tumor microenvironment. Cell Mol Immunol.

[32] Peng D, Kryczek I, Nagarsheth N, Zhao L, Wei S, Wang W (2015). Epigenetic silencing of TH1-type chemokines shapes tumour immunity and immunotherapy. Nature.

[33] Mazzone R, Zwergel C, Mai A, Valente S (2017). Epi-drugs in combination with immunotherapy: a new avenue to improve anticancer efficacy. Clin Epigenet.

[34] Baitsch L, Baumgaertner P, Devêvre E, Raghav SK, Legat A, Barba L (2011). Exhaustion of tumor-specific CD8^+^ T cells in metastases from melanoma patients. J Clin Invest.

[35] Fourcade J, Sun Z, Benallaoua M, Guillaume P, Luescher IF, Sander C (2010). Upregulation of Tim-3 and PD-1 expression is associated with tumor antigen-specific CD8^+^ T cell dysfunction in melanoma patients. J Exp Med.

[36] Sakuishi K, Apetoh L, Sullivan JM, Blazar BR, Kuchroo VK, Anderson AC (2010). Targeting Tim-3 and PD-1 pathways to reverse T cell exhaustion and restore anti-tumor immunity. J Exp Med.

[37] Moskophidis D, Lechner F, Pircher H, Zinkernagel RM (1993). Virus persistence in acutely infected immunocompetent mice by exhaustion of antiviral cytotoxic effector T cells. Nature.

[38] Zehn D, Thimme R, Lugli E, de Almeida GP, Oxenius A (2022). 'Stem-like' precursors are the fount to sustain persistent CD8^+^ T cell responses. Nat Immunol.

[39] Xing C, Li H, Li RJ, Yin L, Zhang HF, Huang ZN (2021). The roles of exosomal immune checkpoint proteins in tumors. Mil Med Res.

[40] Yost KE, Satpathy AT, Wells DK, Qi Y, Wang C, Kageyama R (2019). Clonal replacement of tumor-specific T cells following PD-1 blockade. Nat Med.

[41] Giles JR, Ngiow SF, Manne S, Baxter AE, Khan O, Wang P (2022). Shared and distinct biological circuits in effector, memory and exhausted CD8^+^ T cells revealed by temporal single-cell transcriptomics and epigenetics. Nat Immunol.

[42] Galletti G, de Simone G, Mazza EMC, Puccio S, Mezzanotte C, Bi TM (2020). Two subsets of stem-like CD8^+^ memory T cell progenitors with distinct fate commitments in humans. Nat Immunol.

[43] Beltra JC, Manne S, Abdel-Hakeem MS, Kurachi M, Giles JR, Chen Z (2020). Developmental relationships of four exhausted CD8^+^ T cell subsets reveals underlying transcriptional and epigenetic landscape control mechanisms. Immunity.

[44] Zhang Y, Chen H, Mo H, Hu X, Gao R, Zhao Y (2021). Single-cell analyses reveal key immune cell subsets associated with response to PD-L1 blockade in triple-negative breast cancer. Cancer Cell.

[45] Wu TD, Madireddi S, de Almeida PE, Banchereau R, Chen YJJ, Chitre AS (2020). Peripheral T cell expansion predicts tumour infiltration and clinical response. Nature.

[46] Sade-Feldman M, Yizhak K, Bjorgaard SL, Ray JP, de Boer CG, Jenkins RW (2018). Defining T cell states associated with response to checkpoint immunotherapy in melanoma. Cell.

[47] Gueguen P, Metoikidou C, Dupic T, Lawand M, Goudot C, Baulande S (2021). Contribution of resident and circulating precursors to tumor-infiltrating CD8^+^ T cell populations in lung cancer. Sci Immunol..

[48] Liu B, Hu X, Feng K, Gao R, Xue Z, Zhang S (2022). Temporal single-cell tracing reveals clonal revival and expansion of precursor exhausted T cells during anti-PD-1 therapy in lung cancer. Nat Cancer.

[49] Klemm SL, Shipony Z, Greenleaf WJ (2019). Chromatin accessibility and the regulatory epigenome. Nat Rev Genet.

[50] Budziszewski GR, Zhao Y, Spangler CJ, Kedziora KM, Williams MR, Azzam DN (2022). Multivalent DNA and nucleosome acidic patch interactions specify VRK1 mitotic localization and activity. Nucleic Acids Res.

[51] Lee CK, Shibata Y, Rao B, Strahl BD, Lieb JD (2004). Evidence for nucleosome depletion at active regulatory regions genome-wide. Nat Genet.

[52] Clapier CR, Iwasa J, Cairns BR, Peterson CL (2017). Mechanisms of action and regulation of ATP-dependent chromatin-remodelling complexes. Nat Rev Mol Cell Biol.

[53] Rao SSP, Huntley MH, Durand NC, Stamenova EK, Bochkov ID, Robinson JT (2014). A 3D map of the human genome at kilobase resolution reveals principles of chromatin looping. Cell.

[54] MacRae TA, Fothergill-Robinson J, Ramalho-Santos M (2023). Regulation, functions and transmission of bivalent chromatin during mammalian development. Nat Rev Mol Cell Biol.

[55] Lee JS, Smith E, Shilatifard A (2010). The language of histone crosstalk. Cell.

[56] Turner BM (2007). Defining an epigenetic code. Nat Cell Biol.

[57] Singh M, Kumar V, Sehrawat N, Yadav M, Chaudhary M, Upadhyay SK (2022). Current paradigms in epigenetic anticancer therapeutics and future challenges. Semin Cancer Biol.

[58] Waitkus MS, Diplas BH, Yan H (2018). Biological role and therapeutic potential of IDH mutations in cancer. Cancer Cell.

[59] Gräff J, Tsai LH (2013). Histone acetylation: molecular mnemonics on the chromatin. Nat Rev Neurosci.

[60] Bondarev AD, Attwood MM, Jonsson J, Chubarev VN, Tarasov VV, Schiöth HB (2021). Recent developments of HDAC inhibitors: emerging indications and novel molecules. Br J Clin Pharmacol.

[61] Popat R, Brown SR, Flanagan L, Hall A, Gregory W, Kishore B (2016). Bortezomib, thalidomide, dexamethasone, and panobinostat for patients with relapsed multiple myeloma (MUK-six): a multicentre, open-label, phase 1/2 trial. Lancet Haematol.

[62] Rambaldi A, Iurlo A, Vannucchi AM, Martino B, Guarini A, Ruggeri M (2021). Long-term safety and efficacy of givinostat in polycythemia vera: 4-year mean follow up of three phase 1/2 studies and a compassionate use program. Blood Cancer J.

[63] An Z, Lv W, Su S, Wu W, Rao Y (2019). Developing potent PROTACs tools for selective degradation of HDAC6 protein. Protein Cell.

[64] Yang K, Wu H, Zhang Z, Leisten ED, Nie X, Liu B (2020). Development of selective histone deacetylase 6 (HDAC6) degraders recruiting von hippel-lindau (VHL) E3 ubiquitin ligase. ACS Med Chem Lett.

[65] Manni W, Jianxin X, Weiqi H, Siyuan C, Huashan S (2022). JMJD family proteins in cancer and inflammation. Signal Transduct Target Ther.

[66] Zhang L, Chen Y, Li Z, Lin C, Zhang T, Wang G (2023). Development of JmjC-domain-containing histone demethylase (KDM2-7) inhibitors for cancer therapy. Drug Discov Today.

[67] Belk JA, Yao W, Ly N, Freitas KA, Chen Y-T, Shi Q (2022). Genome-wide CRISPR screens of T cell exhaustion identify chromatin remodeling factors that limit T cell persistence. Cancer Cell.

[68] Gennert DG, Lynn RC, Granja JM, Weber EW, Mumbach MR, Zhao Y (2021). Dynamic chromatin regulatory landscape of human CAR T cell exhaustion. Proc Natl Acad Sci U S A.

[69] Farnaby W, Koegl M, Roy MJ, Whitworth C, Diers E, Trainor N (2019). BAF complex vulnerabilities in cancer demonstrated via structure-based PROTAC design. Nat Chem Biol.

[70] Marian CA, Stoszko M, Wang L, Leighty MW, de Crignis E, Maschinot CA (2018). Small molecule targeting of specific BAF (mSWI/SNF) complexes for HIV latency reversal. Cell Chem Biol.

[71] Brien GL, Stegmaier K, Armstrong SA (2019). Targeting chromatin complexes in fusion protein-driven malignancies. Nat Rev Cancer.

[72] Wimalasena VK, Wang T, Sigua LH, Durbin AD, Qi J (2020). Using chemical epigenetics to target cancer. Mol Cell.

[73] Holoch D, Moazed D (2015). RNA-mediated epigenetic regulation of gene expression. Nat Rev Genet.

[74] Gutbrod MJ, Martienssen RA (2020). Conserved chromosomal functions of RNA interference. Nat Rev Genet.

[75] Martienssen R, Moazed D (2015). RNAi and heterochromatin assembly. Cold Spring Harb Perspect Biol.

[76] Moffett HF, Cartwright ANR, Kim HJ, Godec J, Pyrdol J, Äijö T (2017). The microRNA miR-31 inhibits CD8^+^ T cell function in chronic viral infection. Nat Immunol.

[77] Stelekati E, Chen Z, Manne S, Kurachi M, Ali MA, Lewy K (2018). Long-term persistence of exhausted CD8 T cells in chronic infection is regulated by microRNA-155. Cell Rep.

[78] Stelekati E, Cai Z, Manne S, Chen Z, Beltra JC, Buchness LA (2022). MicroRNA-29a attenuates CD8 T cell exhaustion and induces memory-like CD8 T cells during chronic infection. Proc Natl Acad Sci U S A.

[79] Chen LL (2020). The expanding regulatory mechanisms and cellular functions of circular RNAs. Nat Rev Mol Cell Biol.

[80] Liu CX, Chen LL (2022). Circular RNAs: characterization, cellular roles, and applications. Cell.

[81] Zhang LX, Gao J, Long X, Zhang PF, Yang X, Zhu SQ (2022). The circular RNA circHMGB2 drives immunosuppression and anti-PD-1 resistance in lung adenocarcinomas and squamous cell carcinomas via the miR-181a-5p/CARM1 axis. Mol Cancer.

[82] Wei CY, Zhu MX, Lu NH, Liu JQ, Yang YW, Zhang Y (2020). Circular RNA circ_0020710 drives tumor progression and immune evasion by regulating the miR-370-3p/CXCL12 axis in melanoma. Mol Cancer.

[83] Yang C, Wu S, Mou Z, Zhou Q, Dai X, Ou Y (2022). Exosome-derived circTRPS1 promotes malignant phenotype and CD8^+^ T cell exhaustion in bladder cancer microenvironments. Mol Ther.

[84] Wang J, Zhao X, Wang Y, Ren F, Sun D, Yan Y (2020). circRNA-002178 act as a ceRNA to promote PDL1/PD1 expression in lung adenocarcinoma. Cell Death Dis.

[85] Liu Z, Wang T, She Y, Wu K, Gu S, Li L (2021). N^6^-methyladenosine-modified circIGF_2_BP_3_ inhibits CD8+ T-cell responses to facilitate tumor immune evasion by promoting the deubiquitination of PD-L1 in non-small cell lung cancer. Mol Cancer.

[86] Ge J, Wang J, Xiong F, Jiang X, Zhu K, Wang Y (2021). Epstein-Barr virus-encoded circular RNA circBART2.2 promotes immune escape of nasopharyngeal carcinoma by regulating PD-L1. Cancer Res..

[87] Fu X, Sun G, Tu S, Fang K, Xiong Y, Tu Y (2022). Hsa_circ_0046523 mediates an immunosuppressive tumor microenvironment by regulating miR-148a-3p/PD-L1 axis in pancreatic cancer. Front Oncol.

[88] Huang XY, Zhang PF, Wei CY, Peng R, Lu JC, Gao C (2020). Circular RNA circMET drives immunosuppression and anti-PD1 therapy resistance in hepatocellular carcinoma via the miR-30-5p/snail/DPP4 axis. Mol Cancer.

[89] Sun H, Li K, Liu C, Yi C (2023). Regulation and functions of non-m^6^A mRNA modifications. Nat Rev Mol Cell Biol.

[90] Deng X, Qing Y, Horne D, Huang H, Chen J (2023). The roles and implications of RNA m^6^A modification in cancer. Nat Rev Clin Oncol.

[91] Liu Y, Zhou J, Li X, Zhang X, Shi J, Wang X (2022). tRNA-m1A modification promotes T cell expansion via efficient MYC protein synthesis. Nat Immunol.

[92] Childs-Disney JL, Yang X, Gibaut QMR, Tong Y, Batey RT, Disney MD (2022). Targeting RNA structures with small molecules. Nat Rev Drug Discov.

[93] Pandey SK, Wheeler TM, Justice SL, Kim A, Younis HS, Gattis D (2015). Identification and characterization of modified antisense oligonucleotides targeting DMPK in mice and nonhuman primates for the treatment of myotonic dystrophy type 1. J Pharmacol Exp Ther.

[94] Matsui M, Corey DR (2017). Non-coding RNAs as drug targets. Nat Rev Drug Discov.

[95] Maucort C, Vo DD, Aouad S, Charrat C, Azoulay S, Di Giorgio A (2021). Design and implementation of synthetic RNA binders for the inhibition of miR-21 biogenesis. ACS Med Chem Lett.

[96] Costales MG, Aikawa H, Li Y, Childs-Disney JL, Abegg D, Hoch DG (2020). Small-molecule targeted recruitment of a nuclease to cleave an oncogenic RNA in a mouse model of metastatic cancer. Proc Natl Acad Sci U S A.

[97] Zhang P, Liu X, Abegg D, Tanaka T, Tong Y, Benhamou RI (2021). Reprogramming of protein-targeted small-molecule medicines to RNA by ribonuclease recruitment. J Am Chem Soc.

[98] Argilés JM, López-Soriano FJ, Stemmler B, Busquets S (2023). Cancer-associated cachexia - understanding the tumour macroenvironment and microenvironment to improve management. Nat Rev Clin Oncol.

[99] Wang YY, Wu ZZ, Huang CF, Sun ZJ (2023). Tumor-host colluding through erythroid progenitor cells: Mechanisms and opportunities. Cancer Lett.

[100] Tien FM, Lu HH, Lin SY, Tsai HC (2023). Epigenetic remodeling of the immune landscape in cancer: therapeutic hurdles and opportunities. J Biomed Sci.

[101] Gomez S, Tabernacki T, Kobyra J, Roberts P, Chiappinelli KB (2020). Combining epigenetic and immune therapy to overcome cancer resistance. Semin Cancer Biol.

[102] Lin JH, Huffman AP, Wattenberg MM, Walter DM, Carpenter EL, Feldser DM (2020). Type 1 conventional dendritic cells are systemically dysregulated early in pancreatic carcinogenesis. J Exp Med.

[103] Spitzer MH, Carmi Y, Reticker-Flynn NE, Kwek SS, Madhireddy D, Martins MM (2017). Systemic immunity is required for effective cancer immunotherapy. Cell.

[104] Dähling S, Mansilla AM, Knöpper K, Grafen A, Utzschneider DT, Ugur M (2022). Type 1 conventional dendritic cells maintain and guide the differentiation of precursors of exhausted T cells in distinct cellular niches. Immunity.

[105] Morrison AH, Diamond MS, Hay CA, Byrne KT, Vonderheide RH (2020). Sufficiency of CD40 activation and immune checkpoint blockade for T cell priming and tumor immunity. Proc Natl Acad Sci U S A.

[106] Corrales L, Glickman LH, McWhirter SM, Kanne DB, Sivick KE, Katibah GE (2015). Direct activation of STING in the tumor microenvironment leads to potent and systemic tumor regression and immunity. Cell Rep.

[107] Ohkuri T, Kosaka A, Ishibashi K, Kumai T, Hirata Y, Ohara K (2017). Intratumoral administration of cGAMP transiently accumulates potent macrophages for anti-tumor immunity at a mouse tumor site. Cancer Immunol Immunother.

[108] Spranger S, Dai D, Horton B, Gajewski TF (2017). Tumor-residing Batf3 dendritic cells are required for effector T cell trafficking and adoptive T cell therapy. Cancer Cell.

[109] Kerdidani D, Chouvardas P, Arjo AR, Giopanou I, Ntaliarda G, Guo YA (2019). Wnt1 silences chemokine genes in dendritic cells and induces adaptive immune resistance in lung adenocarcinoma. Nat Commun.

[110] Nagarsheth N, Peng D, Kryczek I, Wu K, Li W, Zhao E (2016). PRC2 epigenetically silences Th1-type chemokines to suppress effector T-cell trafficking in Colon cancer. Cancer Res.

[111] Zheng H, Zhao W, Yan C, Watson CC, Massengill M, Xie M (2016). HDAC inhibitors enhance T-cell chemokine expression and augment response to PD-1 immunotherapy in lung adenocarcinoma. Clin Cancer Res.

[112] Topper MJ, Vaz M, Chiappinelli KB, DeStefano Shields CE, Niknafs N, Yen RWC (2017). Epigenetic therapy ties MYC depletion to reversing immune evasion and treating lung cancer. Cell..

[113] Brocks D, Schmidt CR, Daskalakis M, Jang HS, Shah NM, Li D (2017). DNMT and HDAC inhibitors induce cryptic transcription start sites encoded in long terminal repeats. Nat Genet.

[114] Ozga AJ, Chow MT, Luster AD (2021). Chemokines and the immune response to cancer. Immunity.

[115] Rodriques SG, Stickels RR, Goeva A, Martin CA, Murray E, Vanderburg CR (2019). Slide-seq: a scalable technology for measuring genome-wide expression at high spatial resolution. Science.

[116] Goltsev Y, Samusik N, Kennedy-Darling J, Bhate S, Hale M, Vazquez G (2018). Deep profiling of mouse splenic architecture with CODEX multiplexed imaging. Cell.

[117] Thommen DS, Koelzer VH, Herzig P, Roller A, Trefny M, Dimeloe S (2018). A transcriptionally and functionally distinct PD-1^+^ CD8^+^ T cell pool with predictive potential in non-small-cell lung cancer treated with PD-1 blockade. Nat Med.

[118] Minnar CM, Chariou PL, Horn LA, Hicks KC, Palena C, Schlom J (2022). Tumor-targeted interleukin-12 synergizes with entinostat to overcome PD-1/PD-L1 blockade-resistant tumors harboring MHC-I and APM deficiencies. J Immunother Cancer.

[119] Sautès-Fridman C, Petitprez F, Calderaro J, Fridman WH (2019). Tertiary lymphoid structures in the era of cancer immunotherapy. Nat Rev Cancer.

[120] Siddiqui I, Schaeuble K, Chennupati V, Fuertes Marraco SA, Calderon-Copete S, Pais Ferreira D (2019). Intratumoral Tcf1^+^PD-1^+^CD8^+^ T cells with stem-like properties promote tumor control in response to vaccination and checkpoint blockade immunotherapy. Immunity.

[121] Duraiswamy J, Turrini R, Minasyan A, Barras D, Crespo I, Grimm AJ (2021). Myeloid antigen-presenting cell niches sustain antitumor T cells and license PD-1 blockade via CD28 costimulation. Cancer Cell.

[122] Eberhardt CS, Kissick HT, Patel MR, Cardenas MA, Prokhnevska N, Obeng RC (2021). Functional HPV-specific PD-1^+^ stem-like CD8 T cells in head and neck cancer. Nature.

[123] Duckworth BC, Lafouresse F, Wimmer VC, Broomfield BJ, Dalit L, Alexandre YO (2021). Effector and stem-like memory cell fates are imprinted in distinct lymph node niches directed by CXCR3 ligands. Nat Immunol.

[124] Dersh D, Phelan JD, Gumina ME, Wang B, Arbuckle JH, Holly J (2021). Genome-wide screens identify lineage- and tumor-specific genes modulating MHC-I- and MHC-II-restricted immunosurveillance of human lymphomas. Immunity.

[125] Loo Yau H, Ettayebi I, De Carvalho DD (2019). The cancer epigenome: exploiting its vulnerabilities for immunotherapy. Trends Cell Biol.

[126] Finn OJ (2018). The dawn of vaccines for cancer prevention. Nat Rev Immunol.

[127] Shimasaki N, Jain A, Campana D (2020). NK cells for cancer immunotherapy. Nat Rev Drug Discov.

[128] Zhang X, Zhang H, Lan H, Wu J, Xiao Y (2023). CAR-T cell therapy in multiple myeloma: current limitations and potential strategies. Front Immunol.

[129] Fukumoto T, Fatkhutdinov N, Zundell JA, Tcyganov EN, Nacarelli T, Karakashev S (2019). HDAC6 inhibition synergizes with anti-PD-L1 therapy in ARID1A-inactivated ovarian cancer. Cancer Res.

[130] Zhu Y, An X, Zhang X, Qiao Y, Zheng T, Li X (2019). STING: a master regulator in the cancer-immunity cycle. Mol Cancer.

[131] Falahat R, Berglund A, Putney RM, Perez-Villarroel P, Aoyama S, Pilon-Thomas S (2021). Epigenetic reprogramming of tumor cell-intrinsic STING function sculpts antigenicity and T cell recognition of melanoma. Proc Natl Acad Sci U S A.

[132] Low JT, Chandramohan V, Bowie ML, Brown MC, Waitkus MS, Briley A (2022). Epigenetic STING silencing is developmentally conserved in gliomas and can be rescued by methyltransferase inhibition. Cancer Cell.

[133] Gao Y, You M, Fu J, Tian M, Zhong X, Du C (2022). Intratumoral stem-like CCR4^+^ regulatory T cells orchestrate the immunosuppressive microenvironment in HCC associated with hepatitis B. J Hepatol.

[134] Strauss L, Mahmoud MAA, Weaver JD, Tijaro-Ovalle NM, Christofides A, Wang Q (2020). Targeted deletion of PD-1 in myeloid cells induces antitumor immunity. Sci Immunol..

[135] Rodríguez-Ubreva J, Català-Moll F, Obermajer N, Álvarez-Errico D, Ramirez RN, Company C (2017). Prostaglandin E_2_ leads to the acquisition of DNMT3A-dependent tolerogenic functions in human myeloid-derived suppressor cells. Cell Rep.

[136] Romine KA, MacPherson K, Cho HJ (2023). BET inhibitors rescue anti-PD1 resistance by enhancing TCF7 accessibility in leukemia-derived terminally exhausted CD8^+^ T cells. Leukemia.

[137] Sasidharan Nair V, Saleh R, Toor SM, Taha RZ, Ahmed AA, Kurer MA (2020). Transcriptomic profiling disclosed the role of DNA methylation and histone modifications in tumor-infiltrating myeloid-derived suppressor cell subsets in colorectal cancer. Clin Epigenet.

[138] Izumi Y, Kanayama M, Shen Z, Kai M, Kawamura S, Akiyama M (2021). An antibody-drug conjugate that selectively targets human monocyte progenitors for anti-cancer therapy. Front Immunol.

[139] Li B, Sun S, Li JJ, Yuan JP, Sun SR, Wu Q (2023). Adipose tissue macrophages: implications for obesity-associated cancer. Mil Med Res.

[140] Chen S, Yang J, Wei Y, Wei X (2020). Epigenetic regulation of macrophages: from homeostasis maintenance to host defense. Cell Mol Immunol.

[141] Maier B, Leader AM, Chen ST, Tung N, Chang C, LeBerichel J (2020). A conserved dendritic-cell regulatory program limits antitumour immunity. Nature.

[142] Kvedaraite E, Ginhoux F (2022). Human dendritic cells in cancer. Sci Immunol..

[143] Mendes K, Schmidhofer S, Minderjahn J, Glatz D, Kiesewetter C, Raithel J (2021). The epigenetic pioneer EGR2 initiates DNA demethylation in differentiating monocytes at both stable and transient binding sites. Nat Commun.

[144] Pacis A, Mailhot-Léonard F, Tailleux L, Randolph HE, Yotova V, Dumaine A (2019). Gene activation precedes DNA demethylation in response to infection in human dendritic cells. Proc Natl Acad Sci U S A.

[145] Kumagai S, Togashi Y, Kamada T, Sugiyama E, Nishinakamura H, Takeuchi Y (2020). The PD-1 expression balance between effector and regulatory T cells predicts the clinical efficacy of PD-1 blockade therapies. Nat Immunol.

[146] Kamada T, Togashi Y, Tay C, Ha D, Sasaki A, Nakamura Y (2019). PD-1^+^ regulatory T cells amplified by PD-1 blockade promote hyperprogression of cancer. Proc Natl Acad Sci U S A.

[147] Kawakami R, Kitagawa Y, Chen KY, Arai M, Ohara D, Nakamura Y (2021). Distinct Foxp3 enhancer elements coordinate development, maintenance, and function of regulatory T cells. Immunity.

[148] Cameron J, Martino P, Nguyen L, Li X (2020). Cutting edge: CRISPR-based transcriptional regulators reveal transcription-dependent establishment of epigenetic memory of Foxp3 in regulatory T cells. J Immunol.

[149] Kwon HK, Chen HM, Mathis D, Benoist C (2017). Different molecular complexes that mediate transcriptional induction and repression by FoxP3. Nat Immunol.

[150] Costantini B, Kordasti SY, Kulasekararaj AG, Jiang J, Seidl T, Abellan PP (2013). The effects of 5-azacytidine on the function and number of regulatory T cells and T-effectors in myelodysplastic syndrome. Haematologica.

[151] Stübig T, Badbaran A, Luetkens T, Hildebrandt Y, Atanackovic D, Binder TMC (2014). 5-azacytidine promotes an inhibitory T-cell phenotype and impairs immune mediated antileukemic activity. Mediators Inflamm.

[152] Kröger N, Sockel K, Wolschke C, Bethge W, Schlenk RF, Wolf D (2021). Comparison between 5-azacytidine treatment and allogeneic stem-cell transplantation in elderly patients with advanced MDS according to donor availability (VidazaAllo study). J Clin Oncol.

[153] Montesinos P, Recher C, Vives S, Zarzycka E, Wang J, Bertani G (2022). Ivosidenib and azacitidine in IDH1-mutated acute myeloid leukemia. N Engl J Med.

[154] Huang J, Wang L, Dahiya S, Beier UH, Han R, Samanta A (2017). Histone/protein deacetylase 11 targeting promotes Foxp3^+^ Treg function. Sci Rep.

[155] Tao R, de Zoeten EF, Ozkaynak E, Chen C, Wang L, Porrett PM (2007). Deacetylase inhibition promotes the generation and function of regulatory T cells. Nat Med.

[156] Xiao H, Jiao J, Wang L, O’Brien S, Newick K, Wang LCS (2016). HDAC5 controls the functions of Foxp3^+^ T-regulatory and CD8^+^ T cells. Int J Cancer..

[157] Castillo J, Wu E, Lowe C, Srinivasan S, McCord R, Wagle M-C (2019). CBP/p300 drives the differentiation of regulatory T cells through transcriptional and non-transcriptional mechanisms. Cancer Res.

[158] Zhang Q, Fang Y, Lv C (2022). Norisoboldine induces the development of Treg cells by promoting fatty acid oxidation-mediated H3K27 acetylation of Foxp3. FASEB J.

[159] DuPage M, Chopra G, Quiros J, Rosenthal WL, Morar MM, Holohan D (2015). The chromatin-modifying enzyme Ezh2 is critical for the maintenance of regulatory T cell identity after activation. Immunity.

[160] Wang D, Quiros J, Mahuron K, Pai CC, Ranzani V, Young A (2018). Targeting EZH2 reprograms intratumoral regulatory T cells to enhance cancer immunity. Cell Rep.

[161] Northend M, Townsend W (2021). Novel therapy approaches to follicular lymphoma. Drugs.

[162] Xiao Q, Zhou D, Rucki AA, Williams J, Zhou J, Mo G (2016). Cancer-associated fibroblasts in pancreatic cancer are reprogrammed by tumor-induced alterations in genomic DNA methylation. Cancer Res.

[163] Albrengues J, Bertero T, Grasset E, Bonan S, Maiel M, Bourget I (2015). Epigenetic switch drives the conversion of fibroblasts into proinvasive cancer-associated fibroblasts. Nat Commun.

[164] Li A, Chen P, Leng Y, Kang J (2018). Histone deacetylase 6 regulates the immunosuppressive properties of cancer-associated fibroblasts in breast cancer through the STAT3-COX2-dependent pathway. Oncogene.

[165] Zong Y, Huang J, Sankarasharma D, Morikawa T, Fukayama M, Epstein JI (2012). Stromal epigenetic dysregulation is sufficient to initiate mouse prostate cancer via paracrine Wnt signaling. Proc Natl Acad Sci U S A.

[166] Bhagat TD, von Ahrens D, Dawlaty M, Zou Y, Baddour J, Achreja A (2019). Lactate-mediated epigenetic reprogramming regulates formation of human pancreatic cancer-associated fibroblasts. Elife.

[167] Grzywa TM, Justyniarska M, Nowis D, Golab J (2021). Tumor immune evasion induced by dysregulation of erythroid progenitor cells development. Cancers (Basel).

[168] Sano Y, Yoshida T, Choo M-K, Jiménez-Andrade Y, Hill KR, Georgopoulos K (2021). Multiorgan signaling mobilizes tumor-associated erythroid cells expressing immune checkpoint molecules. Mol Cancer Res.

[169] Zhao L, He R, Long H, Guo B, Jia Q, Qin D (2018). Late-stage tumors induce anemia and immunosuppressive extramedullary erythroid progenitor cells. Nat Med.

[170] Han Y, Liu Q, Hou J, Gu Y, Zhang Y, Chen Z (2018). Tumor-induced generation of splenic erythroblast-like Ter-cells promotes tumor progression. Cell.

[171] Long H, Jia Q, Wang L, Fang W, Wang Z, Jiang T (2022). Tumor-induced erythroid precursor-differentiated myeloid cells mediate immunosuppression and curtail anti-PD-1/PD-L1 treatment efficacy. Cancer Cell.

[172] Schulz VP, Yan H, Lezon-Geyda K, An X, Hale J, Hillyer CD (2019). A unique epigenomic landscape defines human erythropoiesis. Cell Rep.

[173] Ludwig LS, Lareau CA, Bao EL, Nandakumar SK, Muus C, Ulirsch JC (2019). Transcriptional states and chromatin accessibility underlying human erythropoiesis. Cell Rep.

[174] Yan H, Wang Y, Qu X, Li J, Hale J, Huang Y (2017). Distinct roles for TET family proteins in regulating human erythropoiesis. Blood.

[175] Oudelaar AM, Beagrie RA, Gosden M, de Ornellas S, Georgiades E, Kerry J (2020). Dynamics of the 4D genome during in vivo lineage specification and differentiation. Nat Commun.

[176] Chaurasia P, Berenzon D, Hoffman R (2011). Chromatin-modifying agents promote the ex vivo production of functional human erythroid progenitor cells. Blood.

[177] Hu P, Nebreda AR, Hanenberg H, Kinnebrew GH, Ivan M, Yoder MC (2018). P38α/JNK signaling restrains erythropoiesis by suppressing Ezh2-mediated epigenetic silencing of Bim. Nat Commun.

[178] Yu L, Myers G, Ku C-J, Schneider E, Wang Y, Singh SA (2021). An erythroid-to-myeloid cell fate conversion is elicited by LSD1 inactivation. Blood.

[179] Myers JA, Couch T, Murphy Z, Malik J, Getman M, Steiner LA (2020). The histone methyltransferase Setd8 alters the chromatin landscape and regulates the expression of key transcription factors during erythroid differentiation. Epigenet Chromatin.

[180] Ji P, Yeh V, Ramirez T, Murata-Hori M, Lodish HF (2010). Histone deacetylase 2 is required for chromatin condensation and subsequent enucleation of cultured mouse fetal erythroblasts. Haematologica.

[181] Wang Y, Li W, Schulz VP, Zhao H, Qu X, Qi Q (2021). Impairment of human terminal erythroid differentiation by histone deacetylase 5 deficiency. Blood.

[182] DePeaux K, Delgoffe GM (2021). Metabolic barriers to cancer immunotherapy. Nat Rev Immunol.

[183] Møller SH, Hsueh PC, Yu YR, Zhang L, Ho PC (2022). Metabolic programs tailor T cell immunity in viral infection, cancer, and aging. Cell Metab.

[184] van Acker HH, Ma S, Scolaro T, Kaech SM, Mazzone M (2021). How metabolism bridles cytotoxic CD8^+^ T cells through epigenetic modifications. Trends Immunol.

[185] Qiu J, Villa M, Sanin DE, Buck MD, O’Sullivan D, Ching R (2019). Acetate promotes T cell effector function during glucose restriction. Cell Rep.

[186] Brand A, Singer K, Koehl GE, Kolitzus M, Schoenhammer G, Thiel A (2016). LDHA-associated lactic acid production blunts tumor immunosurveillance by T and NK cells. Cell Metab.

[187] Chatterjee S, Daenthanasanmak A, Chakraborty P, Wyatt MW, Dhar P, Selvam SP (2018). CD38-NAD^+^axis regulates immunotherapeutic anti-tumor T cell response. Cell Metab.

[188] Dong L, Bi Y, Jia A, Yu Q, Wang Y, Wang Y (2020). Crucial role of histone deacetylase SIRT1 in myeloid-derived suppressor cell-mediated reprogramming of CD4^+^ T-cell differentiation. Cell Mol Immunol.

[189] Suzuki J, Yamada T, Inoue K, Nabe S, Kuwahara M, Takemori N (2018). The tumor suppressor menin prevents effector CD8 T-cell dysfunction by targeting mTORC1-dependent metabolic activation. Nat Commun.

[190] Tyrakis PA, Palazon A, Macias D, Lee KL, Phan AT, Veliça P (2016). S-2-hydroxyglutarate regulates CD8^+^ T-lymphocyte fate. Nature.

[191] Bian Y, Li W, Kremer DM, Sajjakulnukit P, Li S, Crespo J (2020). Cancer SLC43A2 alters T cell methionine metabolism and histone methylation. Nature.

[192] Roy DG, Chen J, Mamane V, Ma EH, Muhire BM, Sheldon RD (2020). Methionine metabolism shapes T helper cell responses through regulation of epigenetic reprogramming. Cell Metab.

[193] Jin S, Li M, Chang H, Wang R, Zhang Z, Zhang J (2022). The m^6^A demethylase ALKBH5 promotes tumor progression by inhibiting RIG-I expression and IFN alpha production through the IKKε/TBK1/IRF3 pathway in head and neck squamous cell carcinoma. Mol Cancer.

[194] Vodnala SK, Eil R, Kishton RJ, Sukumar M, Yamamoto TN, Ha NH (2019). T cell stemness and dysfunction in tumors are triggered by a common mechanism. Science..

[195] Scharping NE, Rivadeneira DB, Menk AV, Vignali PDA, Ford BR, Rittenhouse NL (2021). Mitochondrial stress induced by continuous stimulation under hypoxia rapidly drives T cell exhaustion. Nat Immunol.

[196] Vignali PDA, DePeaux K, Watson MJ, Ye C, Ford BR, Lontos K (2023). Hypoxia drives CD39-dependent suppressor function in exhausted T cells to limit antitumor immunity. Nat Immunol.

[197] Hanahan D (2022). Hallmarks of cancer: new dimensions. Cancer Discov.

[198] Yuan S, Fang C, Leng WD, Wu L, Li BH, Wang XH (2021). Oral microbiota in the oral-genitourinary axis: identifying periodontitis as a potential risk of genitourinary cancers. Mil Med Res.

[199] Matson V, Fessler J, Bao R, Chongsuwat T, Zha Y, Alegre ML (2018). The commensal microbiome is associated with anti-PD-1 efficacy in metastatic melanoma patients. Science.

[200] Sivan A, Corrales L, Hubert N, Williams JB, Aquino-Michaels K, Earley ZM (2015). Commensal Bifidobacterium promotes antitumor immunity and facilitates anti-PD-L1 efficacy. Science.

[201] Tanoue T, Morita S, Plichta DR, Skelly AN, Suda W, Sugiura Y (2019). A defined commensal consortium elicits CD8 T cells and anti-cancer immunity. Nature.

[202] Routy B, Le Chatelier E, Derosa L, Duong CPM, Alou MT, Daillère R (2018). Gut microbiome influences efficacy of PD-1-based immunotherapy against epithelial tumors. Science.

[203] Shi Y, Zheng W, Yang K, Harris KG, Ni K, Xue L (2020). Intratumoral accumulation of gut microbiota facilitates CD47-based immunotherapy via STING signaling. J Exp Med.

[204] Overacre-Delgoffe AE, Bumgarner HJ, Cillo AR, Burr AHP, Tometich JT, Bhattacharjee A (2021). Microbiota-specific T follicular helper cells drive tertiary lymphoid structures and anti-tumor immunity against colorectal cancer. Immunity.

[205] Kalaora S, Nagler A, Nejman D, Alon M, Barbolin C, Barnea E (2021). Identification of bacteria-derived HLA-bound peptides in melanoma. Nature.

[206] Haque S, Raina R, Afroze N, Hussain A, Alsulimani A, Singh V (2022). Microbial dysbiosis and epigenetics modulation in cancer development - a chemopreventive approach. Semin Cancer Biol.

[207] Ansari I, Raddatz G, Gutekunst J, Ridnik M, Cohen D, Abu-Remaileh M (2020). The microbiota programs DNA methylation to control intestinal homeostasis and inflammation. Nat Microbiol.

[208] Smith PM, Howitt MR, Panikov N, Michaud M, Gallini CA, Bohlooly-Y M (2013). The microbial metabolites, short-chain fatty acids, regulate colonic Treg cell homeostasis. Science.

[209] Singh RP, Bashir H, Kumar R (2021). Emerging role of microbiota in immunomodulation and cancer immunotherapy. Semin Cancer Biol.

[210] Chow A, Perica K, Klebanoff CA, Wolchok JD (2022). Clinical implications of T cell exhaustion for cancer immunotherapy. Nat Rev Clin Oncol.

[211] Zeidan AM, Boss I, Beach CL, Copeland WB, Thompson E, Fox BA (2022). A randomized phase 2 trial of azacitidine with or without durvalumab as first-line therapy for older patients with AML. Blood Adv.

[212] Zeidan AM, Boss I, Beach CL, Copeland WB, Thompson E, Fox BA (2022). A randomized phase 2 trial of azacitidine with or without durvalumab as first-line therapy for higher-risk myelodysplastic syndromes. Blood Adv.

[213] Kuang C, Park Y, Augustin RC, Lin Y, Hartman DJ, Seigh L (2022). Pembrolizumab plus azacitidine in patients with chemotherapy refractory metastatic colorectal cancer: a single-arm phase 2 trial and correlative biomarker analysis. Clin Epigenet.

[214] Taylor K, Loo Yau H, Chakravarthy A, Wang B, Shen SY, Ettayebi I (2020). An open-label, phase II multicohort study of an oral hypomethylating agent CC-486 and durvalumab in advanced solid tumors. J Immunother Cancer.

[215] Chen S, Xie P, Cowan M, Huang H, Cardenas H, Keathley R (2022). Epigenetic priming enhances antitumor immunity in platinum-resistant ovarian cancer. J Clin Invest.

[216] Daver N, Alotaibi AS, Bücklein V, Subklewe M (2021). T-cell-based immunotherapy of acute myeloid leukemia: current concepts and future developments. Leukemia.

[217] Rutella S, Vadakekolathu J, Mazziotta F, Reeder S, Yau TO, Mukhopadhyay R (2022). Immune dysfunction signatures predict outcomes and define checkpoint blockade-unresponsive microenvironments in acute myeloid leukemia. J Clin Invest.

[218] Fraietta JA, Nobles CL, Sammons MA, Lundh S, Carty SA, Reich TJ (2018). Disruption of TET2 promotes the therapeutic efficacy of CD19-targeted T cells. Nature.

[219] Lynn RC, Weber EW, Sotillo E, Gennert D, Xu P, Good Z (2019). c-Jun overexpression in CAR T cells induces exhaustion resistance. Nature.

[220] Seo H, González-Avalos E, Zhang W, Ramchandani P, Yang C, Lio CWJ (2021). BATF and IRF4 cooperate to counter exhaustion in tumor-infiltrating CAR T cells. Nat Immunol.

[221] Delgoffe GM, Xu C, Mackall CL, Green MR, Gottschalk S, Speiser DE (2021). The role of exhaustion in CAR T cell therapy. Cancer Cell.

[222] Yin X, He L, Guo Z (2023). T-cell exhaustion in CAR-T-cell therapy and strategies to overcome it. Immunology.

[223] Weber EW, Parker KR, Sotillo E, Lynn RC, Anbunathan H, Lattin J (2021). Transient rest restores functionality in exhausted CAR-T cells through epigenetic remodeling. Science..

[224] Yoshikawa T, Wu Z, Inoue S, Kasuya H, Matsushita H, Takahashi Y (2022). Genetic ablation of PRDM1 in antitumor T cells enhances therapeutic efficacy of adoptive immunotherapy. Blood.

[225] Zhu Z, McGray AJR, Jiang W, Lu B, Kalinski P, Guo ZS (2022). Improving cancer immunotherapy by rationally combining oncolytic virus with modulators targeting key signaling pathways. Mol Cancer.

[226] Shalhout SZ, Miller DM, Emerick KS, Kaufman HL (2023). Therapy with oncolytic viruses: progress and challenges. Nat Rev Clin Oncol.

[227] Su M, Pan T, Chen QZ, Zhou WW, Gong Y, Xu G (2022). Data analysis guidelines for single-cell RNA-seq in biomedical studies and clinical applications. Mil Med Res.

[228] Bartosovic M, Kabbe M, Castelo-Branco G (2021). Single-cell CUT&Tag profiles histone modifications and transcription factors in complex tissues. Nat Biotechnol.

[229] Wu SJ, Furlan SN, Mihalas AB, Kaya-Okur HS, Feroze AH, Emerson SN (2021). Single-cell CUT&Tag analysis of chromatin modifications in differentiation and tumor progression. Nat Biotechnol.

[230] Deng Y, Bartosovic M, Kukanja P, Zhang D, Liu Y, Su G (2022). Spatial-CUT&Tag: Spatially resolved chromatin modification profiling at the cellular level. Science.

[231] Deng Y, Bartosovic M, Ma S, Zhang D, Kukanja P, Xiao Y (2022). Spatial profiling of chromatin accessibility in mouse and human tissues. Nature.

[232] Simoni Y, Becht E, Fehlings M, Loh CY, Koo S-L, Teng KWW (2018). Bystander CD8^+^ T cells are abundant and phenotypically distinct in human tumour infiltrates. Nature.

[233] Lin JR, Wang S, Coy S (2023). Multiplexed 3D atlas of state transitions and immune interaction in colorectal cancer. Cell.

[234] Liu Y, Sun L, Zhang H, Shang L, Zhao Y (2021). Microfluidics for drug development: from synthesis to evaluation. Chem Rev.

[235] Schneider P, Walters WP, Plowright AT, Sieroka N, Listgarten J, Goodnow RA (2020). Rethinking drug design in the artificial intelligence era. Nat Rev Drug Discov.

[236] Wang S, Li B, Zhang H, Chen J, Sun X, Xu J (2021). Improving bioavailability of hydrophobic prodrugs through supramolecular nanocarriers based on recombinant proteins for osteosarcoma treatment. Angew Chem Int Ed Engl.

